# First Principles Calculation of Protein–Protein Dimer Affinities of ALS-Associated SOD1 Mutants

**DOI:** 10.3389/fmolb.2022.845013

**Published:** 2022-03-24

**Authors:** Shawn C. C. Hsueh, Mark Nijland, Xubiao Peng, Benjamin Hilton, Steven S. Plotkin

**Affiliations:** ^1^ Department of Physics and Astronomy, University of British Columbia, Vancouver, BC, Canada; ^2^ Laboratory of Organic Chemistry, Wageningen University and Research, Wageningen, Netherlands; ^3^ Laboratory of Physical Chemistry and Soft Matter, Wageningen University and Research, Wageningen, Netherlands; ^4^ Center for Quantum Technology Research, School of Physics, Beijing Institute of Technology, Beijing, China; ^5^ Imperial College London, London, United Kingdom; ^6^ Genome Science and Technology Program, University of British Columbia, Vancouver, BC, Canada

**Keywords:** protein misfolding (conformational) diseases, amyotrophic lateral sclerosis, molecular dynamics simulations, superoxide dismutase (Cu–Zn), dimer dissociation, protein–protein interactions, free energy perturbation, loop entropy

## Abstract

Cu,Zn superoxide dismutase (SOD1) is a 32 kDa homodimer that converts toxic oxygen radicals in neurons to less harmful species. The dimerization of SOD1 is essential to the stability of the protein. Monomerization increases the likelihood of SOD1 misfolding into conformations associated with aggregation, cellular toxicity, and neuronal death in familial amyotrophic lateral sclerosis (fALS). The ubiquity of disease-associated mutations throughout the primary sequence of SOD1 suggests an important role of physicochemical processes, including monomerization of SOD1, in the pathology of the disease. Herein, we use a first-principles statistical mechanics method to systematically calculate the free energy of dimer binding for SOD1 using molecular dynamics, which involves sequentially computing conformational, orientational, and separation distance contributions to the binding free energy. We consider the effects of two ALS-associated mutations in SOD1 protein on dimer stability, A4V and D101N, as well as the role of metal binding and disulfide bond formation. We find that the penalty for dimer formation arising from the conformational entropy of disordered loops in SOD1 is significantly larger than that for other protein–protein interactions previously considered. In the case of the disulfide-reduced protein, this leads to a bound complex whose formation is energetically disfavored. Somewhat surprisingly, the loop free energy penalty upon dimerization is still significant for the holoprotein, despite the increased structural order induced by the bound metal cations. This resulted in a surprisingly modest increase in dimer binding free energy of only about 1.5 kcal/mol upon metalation of the protein, suggesting that the most significant stabilizing effects of metalation are on folding stability rather than dimer binding stability. The mutant A4V has an unstable dimer due to weakened monomer-monomer interactions, which are manifested in the calculation by a separation free energy surface with a lower barrier. The mutant D101N has a stable dimer partially due to an unusually rigid *β*-barrel in the free monomer. D101N also exhibits anticooperativity in loop folding upon dimerization. These computational calculations are, to our knowledge, the most quantitatively accurate calculations of dimer binding stability in SOD1 to date.

## 1 Introduction

Cu,Zn superoxide dismutase (SOD1) is a 32 kDa homodimer that catalyzes the dismutation of oxygen radicals to less harmful species, including molecular oxygen and hydrogen peroxide. Each properly folded monomer in the dimer binds one zinc and one copper atom and contains an intramolecular disulfide bond. Dimerization, metal binding, and disulfide bonding are all important for the stability of the protein, and loss of any of these factors increases the likelihood of SOD1 misfolding into toxic states associated with familial ALS (fALS). SOD1-fALS mutations have been reported to decrease the stability of dimer binding (Doucette et al., 2004; McAlary et al., 2013; Broom et al., 2015a), monomer folding (Lindberg et al., 2002; Lindberg et al., 2005), and metal binding (Tiwari et al., 2009). The variable effects of different mutations on these biophysical components of SOD1 stability have been suggested to be at least partially responsible for the variability in patient survival times in SOD1-related fALS (Lindberg et al., 2005; Wang et al., 2008; Byström et al., 2010; Shi et al., 2016; Abdolvahabi et al., 2017).

In the case of SOD1, over 200 missense, nonsense, frameshift, insertion/deletion, or silent mutational variants dispersed throughout its amino acid sequence have been associated with fALS (http://alsod.iop.kcl.ac.uk) (Wroe et al., 2008). The ubiquity of disease-associated mutations throughout the primary sequence of SOD1 (Andersen, 2000; Valentine et al., 2005) suggests a physicochemical origin for SOD1-fALS, raising the question as to how mutations affect native state quantities, such as dimer stability, metal affinity, disulfide bonding stability, and folding stability, and non-native quantities such misfolded oligomer nucleus size, non-native interaction partners (Huai and Zhang, 2019; Semmler et al., 2020), and propagation speed of aggregates.

In this work, we computationally investigate the effects on the dimer stability of SOD1 due to two fALS mutations and the effects on dimer stability due to disulfide bond reduction. We focus on five mutants/variants of SOD1 protein and calculate their dimer binding free energy from the first principles. The variants and rationale for inclusion in this study are given as follows:

1. WT E,E (SS): Control system for comparison with mutants, computationally straightforward to parameterize. Metal loss increases loop disorder. In a first-principles calculation, we can analyze the penalty due to loop disorder on the binding free energy.
2. WT E,E (SH): Effect of disulfide reduction between C57 and C146 on binding stability. Some studies find that the dimer is unstable (Hörnberg et al., 2007), while others find transiently dimeric populations (Sekhar et al., 2015).
3. A4V E,E (SS): Most common SOD1 fALS mutation in North America, experimentally characterized to decrease stability in the apo state (Byström et al., 2010; Broom et al., 2015b). Binding free energies may be compared with Alchemy calculations (Wells et al., 2021).
4. D101N E,E (SS): Surface residue far from dimer interface, stability comparable to WT SOD1 (Rodriguez et al., 2005; Byström et al., 2010), but reduced Zn affinity, increased protease sensitivity, modest aggregation propensity (Rodriguez et al., 2005), and rapid ALS progression of 2.4 years (Prudencio et al., 2009).
5. WT Cu,Zn (SS): The holoprotein requires reparameterizing the partial charges for the histidines in coordination with Cu and Zn ions (Section 2.3). Cu is taken in the +2 state, and Zn has a charge of +2. The role of metal loss on dimer stability can be specifically investigated by comparing WT Cu,Zn(SS) with WT E,E (SS).

Herein, we calculate dimer binding free energies through all-atom molecular dynamics simulations, using the CHARMM36m potential (Huang et al., 2017) in explicit TIP3P solvent. The improved procedure implemented here follows the formally exact statistical mechanics framework developed by Roux and colleagues and successfully implemented in several smaller proteins (Woo and Roux, 2005; Gumbart et al., 2013a; Gumbart et al., 2013b; Sun et al., 2014; Ulucan et al., 2014; Zeller and Zacharias, 2014; Sayyed-Ahmad et al., 2016; Fu et al., 2017; Lai and Kaznessis, 2017; Prakash et al., 2017; Deng et al., 2018; Zhang et al., 2018; Siebenmorgen and Zacharias, 2019).

The calculation method permits dissection of the contributions to dimer binding free energy. The penalty for dimer formation arising from the conformational entropy of disordered loops in SOD1 is significantly larger than that for other protein–protein interactions previously considered. This necessitated long-time equilibration in replica-exchange umbrella sampling (REMD-US) with appropriately chosen initial seeding to accurately sample the multiple minima present on the potentials of mean force (PMFs). The disulfide bond covalently links loop 4 of SOD1 to the *β*-barrel, and reducing it resulted in sufficient entropy gain to destabilize the dimer in the calculation.

Several observed phenomena are somewhat surprising. Rather than increasing entropy in the bound dimer, disulfide reduction in the apoprotein appears to relieve strain and facilitate increased folding of the loops in the bound dimer. We also found that despite the increased structure induced by the bound metal cations, the relaxation free energy of loops in the holo monomer is nearly as large as that in the apo monomer, leading to a significant loop entropy penalty upon dimerization. This effect partially resulted in a surprisingly modest increase in dimer binding free energy of only about 1.5 kcal/mol more than the apoprotein, suggesting that the most significant effects of metal binding are not on dimer stability but on folding stability. It is worth noting that proper set-up of the holoprotein force field required reparametrizing the metal-coordinating histidines by matching classical and quantum chemical forces and potentials. The apo mutant D101N has a remarkably stable dimer partially due to an unusually rigid *β*-barrel in the free monomer. The mutation D101N also causes a reversal of cooperativity in loop folding upon dimerization. Normally, the ordering of loops in one monomer facilitates the ordering of loops in the other. However, this phenomenon is reversed for this mutation, and ordering loops on one monomer hinders the ordering of loops on the other. The apo A4V mutant has an unstable dimer in our calculations due largely to an allosterically weakened dimer interface and reduced inter-monomeric interactions, which are manifested in the separation distance PMF.

The organization of this article is as follows: In the next section, we describe the theory and computational method yielding the binding free energy, involving a judicious choice of restraint potentials to facilitate step-wise convergence during the calculation. The preparation of reference structures is discussed next, including the quantum chemical reparametrization of metal-coordinating histidines. We next discuss equilibration strategies, the method used to achieve converged PMFs, and the specific conformational, orientational, and angular restraints. The Results section discusses the various contributions that lead to the binding free energies for the 5 SOD1 variants in this study compared with previous experimental results. We finally discuss the implications of our findings and conclude.

## 2 Theory and Methods

### 2.1 Theoretical Calculation of Δ*G*_bind_

Determination of the absolute binding free energy of dimer is carried out using a generalization of the method of Roux and colleagues (Woo and Roux, 2005; Gumbart et al., 2013a) in which a series of restraints are successively applied and released to divide the binding free energy calculation into separate calculations, each with manageable convergence.

**FIGURE 1:**
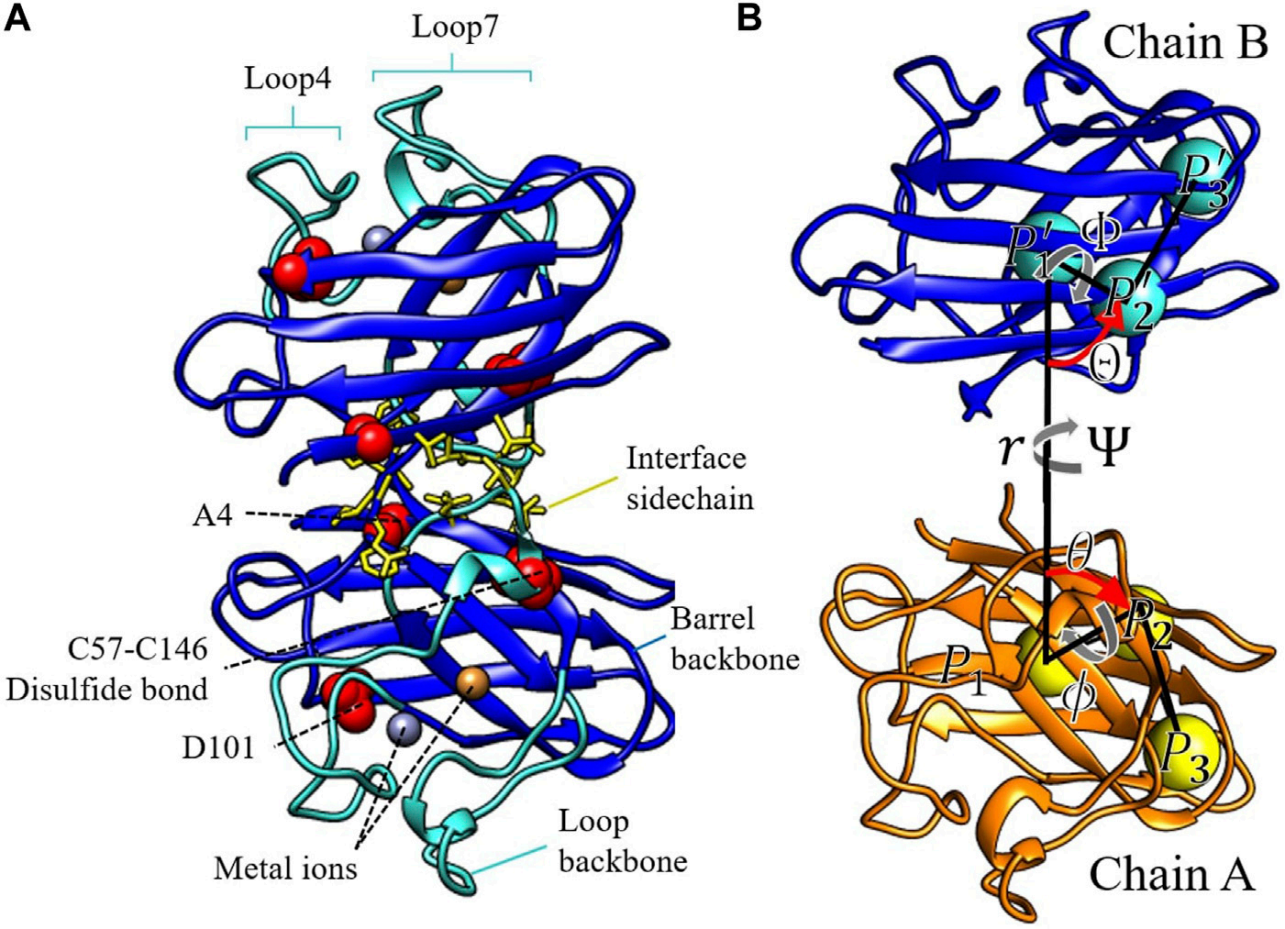
**(A)** The three components of the conformational restraints displayed in color, for the SOD1 homodimer. The central barrel backbone is in blue. The backbones of the large flexible loops 4 and 7 are in turquoise. The side chains of the dimer interface residue are in yellow licorice. The structural elements altered in this study are labeled in panel **(A)** and rendered in red van der Waals spheres. **(B)** Representation of the local reference frame of WT E,E (SS) used to define chain B position and orientation relative to chain A (see text for a description).

The restraints in this study include conformational restraints, orientational restraints, and angular restraints. The restraining potentials are listed in Table 1, along with their values for the protein mutants and variants considered in this study. The conformational restraints restrain the backbone atoms (N, C_*α*_, and C for each residue) of the entire protein and the sidechain atoms of the dimer interface residues (described below). SOD1 contains two long loops, from residues 48–83 (loop 4) and residues 121–143 (loop 7), which contain minimal secondary structure and undergo large conformational rearrangements in the metal-depleted dimer (Elam et al., 2003a; Elam et al., 2003b; Strange et al., 2003; Roberts et al., 2007; Cao et al., 2008; Ahmad et al., 2009; Kevin et al., 2015), apo monomer (Banci et al., 2003; Tom et al., 2009; Das and Plotkin, 2013a), and disulfide-reduced monomer (Das and Plotkin, 2013a; Kumar et al., 2018a) (turquoise in Figures 1A,B).

**TABLE 1:** Free energies associated with the contributions to the binding free energy Δ*G*_bind_.

Contribution	WT E,E (SS)	WT E,E (SH)	A4V E,E (SS)	D101N E,E (SS)	WT Cu,Zn(SS)
	(kcal/mol)	(kcal/mol)	(kcal/mol)	(kcal/mol)	(kcal/mol)
$\Delta G_{LA,c}^{\text{bound}}$	4.08 ± 1.90	1.73 ± 0.51	4.74 ± 0.94	2.73 ± 0.67	0.13 ± 0.01
$\Delta G_{LB,c}^{\text{bound}}$	3.79 ± 0.32	2.63 ± 0.29	2.48 ± 0.25	4.40 ± 0.88	0.21 ± 0.03
$\Delta G_{BA,c}^{\text{bound}}$	1.12 ± 0.11	0.53 ± 0.05	1.36 ± 0.02	0.50 ± 0.02	0.73 ± 0.01
$\Delta G_{BB,c}^{\text{bound}}$	0.43 ± 0.01	1.32 ± 0.06	2.17 ± 0.02	1.90 ± 0.03	0.27 ± 0.03
$\Delta G_{IA,c}^{\text{bound}}$	0.37 ± 0.01	1.65 ± 0.04	0.47 ± 0.02	0.86 ± 0.05	0.49 ± 0.03
$\Delta G_{IB,c}^{\text{bound}}$	0.80 ± 0.10	0.58 ± 0.03	0.21 ± 0.01	0.64 ± 0.19	0.41 ± 0.04
$\Delta G_{\Theta ,o}^{\text{bound}}$	0.45 ± 0.01	0.33 ± 0.02	0.30 ± 0.01	0.66 ± 0.11	0.27 ± 0.01
$\Delta G_{\Phi ,o}^{\text{bound}}$	0.91 ± 0.06	0.67 ± 0.03	0.42 ± 0.05	0.35 ± 0.01	0.36 ± 0.02
$\Delta G_{\Psi ,o}^{\text{bound}}$	0.82 ± 0.12	0.45 ± 0.01	0.61 ± 0.02	0.51 ± 0.01	0.44 ± 0.02
$\Delta G_{\theta ,a}^{\text{bound}}$	0.33 ± 0.01	0.68 ± 0.02	0.29 ± 0.01	0.37 ± 0.04	0.26 ± 0.01
$\Delta G_{\phi ,a}^{\text{bound}}$	0.25 ± 0.01	0.34 ± 0.07	0.37 ± 0.03	0.75 ± 0.13	0.67 ± 0.05
$\Delta G_{\text{dist}+\text{a}}^{\text{restr}}$	−23.26 ± 0.81	−18.81 ± 0.63	−18.97 ± 0.90	−22.69 ± 0.49	−22.29 ± 1.58
$\Delta G_{o}^{\text{free}}$	7.62	7.62	7.63	7.63	7.62
$\Delta G_{I,c}^{\text{free}}$	6.17 ± 0.14	4.31 ± 0.04	4.41 ± 0.07	5.96 ± 0.41	2.85 ± 0.07
$\Delta G_{B,c}^{\text{free}}$	2.70 ± 0.09	2.73 ± 0.05	4.76 ± 0.27	0.67 ± 0.02	0.50 ± 0.05
$\Delta G_{L,c}^{\text{free}}$	3.90 ± 1.39	4.55 ± 0.24	4.34 ± 0.66	4.35 ± 0.27	3.62 ± 1.32
Δ*G*_bind_	−3.45 ± 2.89	1.04 ± 0.94	2.26 ± 1.67	−6.74 ± 1.42	−4.97 ± 2.46

**FIGURE 2:**
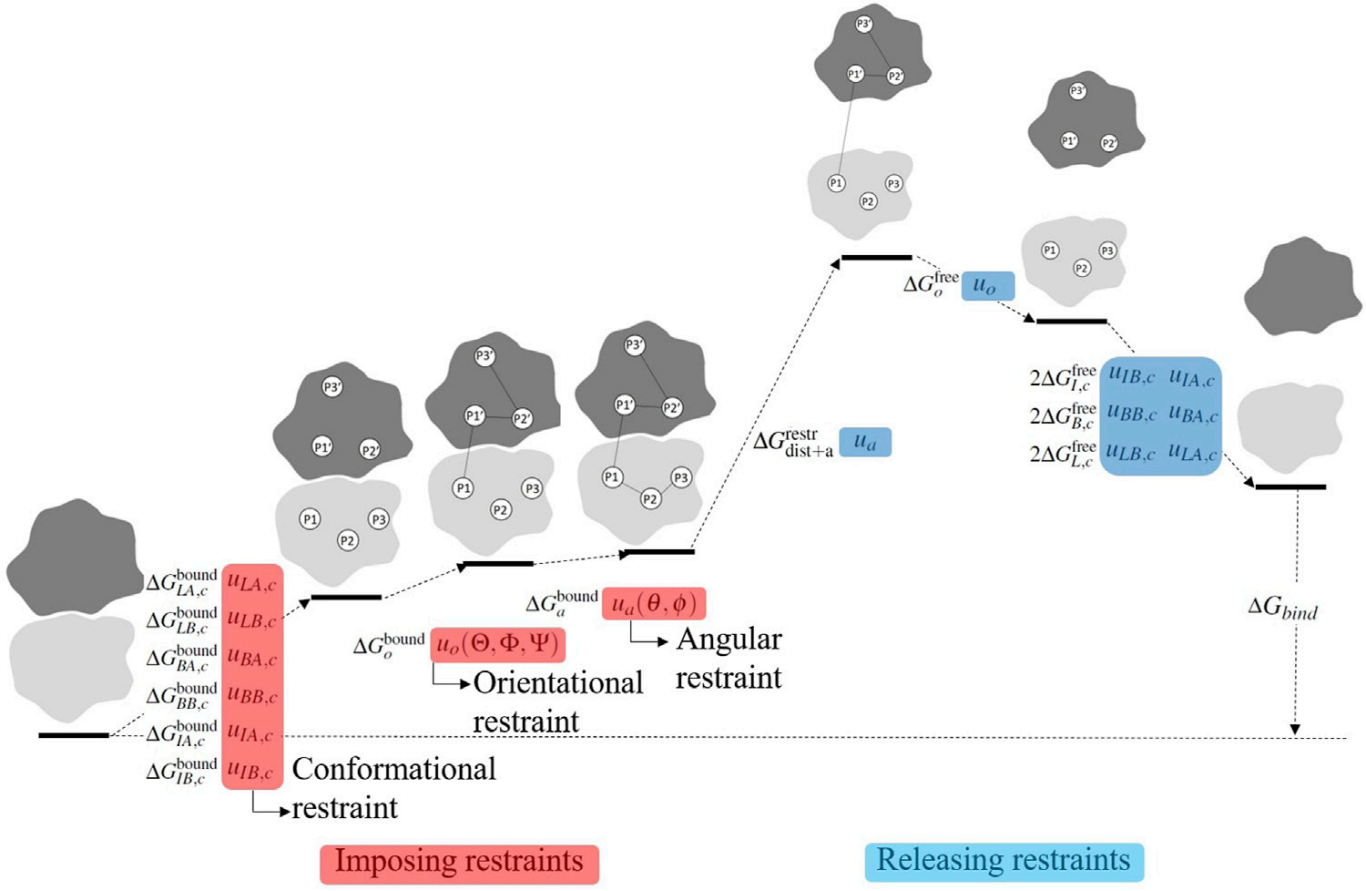
Visualization of the stepwise procedure of calculating the binding free energy Δ*G*_bind_. In order to accelerate convergence, restraints are serially applied in the bound state and serially released in the free state. The full thermodynamic process is given in Eq. 11.

The potentials inducing conformational restraints on backbone atoms of the central barrel in chains A and B of the homodimer (*u*_*BA*,*c*_, *u*_*BB*,*c*_) and the corresponding loops (*u*_*LA*,*c*_, *u*_*LB*,*c*_) are applied separately. The free energies corresponding to applying these restraints to either the bound dimer state (e.g., $\Delta G_{LA,c}^{\text{bound}}$ ) or free state (e.g., $G_{LA,c}^{\text{free}}$ ) are given in Table 1, in the order they are applied (bound state) or released (free state) (see Figure 2). In all cases in Table 1, except for the separation PMF, the free energy is given in terms of applying the restraint (a positive contribution) so that the terms may be simply added to find the binding free energy.

The central barrel consists of residues 1–48, 84–120, and 143–153 (dark blue in Figures 1A,B). The dimer interface sidechain restraint for chains A and B (*u*_*IA*,*c*_, *u*_*IB*,*c*_) is imposed on side chains of residues 5, 7, 50–54, 114, 148, 150–153, which are at least partially buried in the dimer interface in our simulations and are consistent with interface residues reported in previous experimental and theoretical studies (Hough et al., 2004; Das and Plotkin, 2013b) (yellow licorice in Figures 1A,B). The orientational restraint potential (*u*_*o*_) is imposed on Θ, Φ, Ψ angles defined in Figure 1B. The restraint potential *u*_*o*_ (Θ, Φ, Ψ) ensures that the same faces of chain A and chain B point towards each other when calculating other contributions to the dimer binding free energy, such as the potential of mean force (PMF) as a function of separation distance. Similarly, the angular restraint (*u*_*a*_) is imposed on the polar and azimuthal angles *θ* and *ϕ* defined in Figure 1B. The potential *u*_*a*_ (*θ*, *ϕ*) obviates the need to sample the full 4*π* solid angle, whose phase space sampling contribution can be accounted for analytically.

The orientational angles shown in Figure 1B are defined as follows: three groups of atoms are chosen in chain A. *P*_1_ is at the centre of mass of the central beta-barrel structure, represented by residues 1–48, 84–120, and 143–153. *P*_2_ is at the centre of mass of residues 5–7, 17–19, and 32–34, creating a reference point on the surface of the beta sheet of the central barrel. *P*_3_ is at the centre of mass of residues 11–13, 40–42, and 120–122, describing the “lid” of the central beta barrel. Likewise, the same groups of atoms are chosen in chain B to define $P_{1}^{\prime }$ , $P_{2}^{\prime }$ , and $P_{3}^{\prime }$ . The spherical coordinate system establishing the position of chain B relative to chain A is by the distance *r* $\left(\bar{P_{1}P_{1}^{\prime }}\right)$ , angle $\theta \left(∠P_{1}^{\prime }P_{1}P_{2}\right)$ , and the dihedral angle *ϕ* ( $P_{1}^{\prime }$ -*P*_1_-*P*_2_-*P*_3_). The Euler angles needed to define the orientation of chain B relative to chain A as the angle $\Theta \left(∠P_{1}P_{1}^{\prime }P_{2}^{\prime }\right)$ , the dihedral angle Φ (*P*_1_- $P_{1}^{\prime }$ - $P_{2}^{\prime }$ - $P_{3}^{\prime }$ ), and the dihedral angle Ψ (*P*_2_-*P*_1_- $P_{1}^{\prime }$ - $P_{2}^{\prime }$ ). The same definitions for the reference frame apply to all variants.

The absolute binding free energy can be defined in terms of equilibrium binding constant as $\Delta G_{\text{bind}}\equiv -k_{\text{B}}T\ln\left(K_{\text{eq}}c{}^{\circ}\right)$ by assuming a standard state concentration *c*° of 1 mol/L (1 molecule/1661 Å^3^).

The equilibrium constant may be written as a ratio of two integrals, one in the bound state and one in the free state:

$$K_{eq}=\frac{\int_{\text{bound}}dB\int dAe^{-\beta U}}{\int_{\text{free}}dB\delta \left(r_{AB}-r_{AB}^{*}\right)\int dAe^{-\beta U}},$$ (1)

where **A** and **B** correspond to the degrees of freedom of each protein, along with the solvent degrees of freedom that equilibrate about each protein configuration. It is convenient in practice and theoretically justified (Boresch et al., 2003) to use relative coordinates and hold one protein (**A**) fixed while separating the other protein (**B**) from it when calculating the potential of mean force (PMF) and corresponding restraints, as described below.

The essence of the calculation is that the ratio in Eq. 1 may be split by several intermediate integrals involving restraining potentials that effectively multiply the expression by unity but make the thermodynamic averaging tractable (Hermans and Shankar, 1986; Boresch et al., 2003; Woo and Roux, 2005; Gumbart et al., 2013a). The restraining potentials bias the relative orientation, relative position in spherical coordinates, and conformation of each protein to be similar to that in the bound state, as described above. For example, *K*_*eq*_ in Eq. 1 may be written as

$$K_{eq}=\left\{\frac{\int_{\text{bound}}dB\int dAe^{-\beta U}}{\int_{\text{bound}}dB\int dAe^{-\beta \left(U+u_{LA,c}\right)}}\right\}\times \frac{\int_{\text{bound}}dB\int dAe^{-\beta \left(U+u_{LA,c}\right)}}{\int_{\text{free}}dB\delta \left(r_{AB}-r_{AB}^{*}\right)\int dAe^{-\beta U}}\;,$$ (2)

where the first term in curly brackets is a configurational integral equal to ${\left\langle e^{-\beta u_{LA,c}}\right\rangle }_{\left(bound,U\right)}^{-1}$ , which is equal to a free energy difference $e^{\beta \Delta G_{LA,c}^{\text{bound}}}$ that can be calculated using free energy perturbation techniques. With this approach, the equilibrium binding constant *K*_*eq*_ in Eq. 1 can be written as the product of the following free energetic terms:

$$\begin{array}{ll} e^{\beta \Delta G_{LA,c}^{\text{bound}}} & =\frac{\int_{\text{bound}}dB\int dAe^{-\beta U}}{\int_{\text{bound}}dB\int dAe^{-\beta \left(U+u_{LA,c}\right)}} \end{array}$$ (2a)

$$\begin{array}{ll} e^{\beta \Delta G_{LB,c}^{\text{bound}}} & =\frac{\int_{\text{bound}}dB\int dAe^{-\beta \left(U+u_{LA,c}\right)}}{\int_{\text{bound}}dB\int dAe^{-\beta \left(U+u_{LA,c}+u_{LB,c}\right)}} \end{array}$$ (2b)

$$\begin{array}{ll} e^{\beta \Delta G_{BA,c}^{\text{bound}}} & =\frac{\int_{\text{bound}}dB\int dAe^{-\beta \left(U+u_{LA,c}+u_{LB,c}\right)}}{\int_{\text{bound}}dB\int dAe^{-\beta \left(U+u_{LA,c}+u_{LB,c}+u_{BA,c}\right)}} \end{array}$$ (2c)

$$\begin{array}{ll} e^{\beta \Delta G_{BB,c}^{\text{bound}}} & =\frac{\int_{\text{bound}}dB\int dAe^{-\beta \left(U+u_{LA,c}+u_{LB,c}+u_{BA,c}\right)}}{\int_{\text{bound}}dB\int dAe^{-\beta \left(U+u_{LA,c}+u_{LB,c}+u_{BA,c}+u_{BB,c}\right)}} \end{array}$$ (2d)

$$\begin{array}{ll} e^{\beta \Delta G_{IA,c}^{\text{bound}}} & =\frac{\int_{\text{bound}}dB\int dAe^{-\beta \left(U+u_{LA,c}+u_{LB,c}+u_{BA,c}+u_{BB,c}\right)}}{\int_{\text{bound}}dB\int dAe^{-\beta \left(U+u_{LA,c}+u_{LB,c}+u_{BA,c}+u_{BB,c}+u_{IA,c}\right)}} \end{array}$$ (2e)

$$\begin{array}{ll} e^{\beta \Delta G_{IB,c}^{\text{bound}}} & =\frac{\int_{\text{bound}}dB\int dAe^{-\beta \left(U+u_{LA,c}+u_{LB,c}+u_{BA,c}+u_{BB,c}+u_{IA,c}\right)}}{\int_{\text{bound}}dB\int dAe^{-\beta \left(U+u_{LA,c}+u_{LB,c}+u_{BA,c}+u_{BB,c}+u_{IA,c}+u_{IB,c}\right)}} \end{array}$$ (2f)

$$\begin{array}{ll} e^{\beta \Delta G_{o}^{\text{bound}}} & =\frac{\int_{\text{bound}}dB\int dAe^{-\beta \left(U+u_{LA,c}+u_{LB,c}+u_{BA,c}+u_{BB,c}+u_{IA,c}+u_{IB,c}\right)}}{\int_{\text{bound}}dB\int dAe^{-\beta \left(U+u_{LA,c}+u_{LB,c}+u_{BA,c}+u_{BB,c}+u_{IA,c}+u_{IB,c}+u_{o}\right)}} \end{array}$$ (2g)

$$\begin{array}{ll} e^{\beta \Delta G_{a}^{\text{bound}}} & =\frac{\int_{\text{bound}}dB\int dAe^{-\beta \left(U+u_{LA,c}+u_{LB,c}+u_{BA,c}+u_{BB,c}+u_{IA,c}+u_{IB,c}+u_{o}\right)}}{\int_{\text{bound}}dB\int dAe^{-\beta \left(U+u_{LA,c}+u_{LB,c}+u_{BA,c}+u_{BB,c}+u_{IA,c}+u_{IB,c}+u_{o}+u_{a}\right)}} \end{array}$$ (2h)

$$\begin{array}{ll} e^{-\beta \Delta G_{\text{dist}+\text{a}}^{\text{restr}}} & =\frac{c{}^{\circ}\int_{\text{bound}}dB\int dAe^{-\beta \left(U+u_{LA,c}+u_{LB,c}+u_{BA,c}+u_{BB,c}+u_{IA,c}+u_{IB,c}+u_{o}+u_{a}\right)}}{\int_{\text{free}}dB\;\delta \left(r-r^{*}\right)\int dAe^{-\beta \left(U+u_{LA,c}+u_{LB,c}+u_{BA,c}+u_{BB,c}+u_{IA,c}+u_{IB,c}+u_{o}\right)}} \end{array}$$ (2i)

$$\begin{array}{ll} e^{-\beta \Delta G_{o}^{\text{free}}} & =\frac{\int_{\text{free}}dB\;\delta \left(r-r^{*}\right)\int dAe^{-\beta \left(U+u_{LA,c}+u_{LB,c}+u_{BA,c}+u_{BB,c}+u_{IA,c}+u_{IB,c}+u_{o}\right.})}{\int_{\text{free}}dB\;\delta \left(r-r^{*}\right)\int dAe^{-\beta \left(U+u_{LA,c}+u_{LB,c}+u_{BA,c}+u_{BB,c}+u_{IA,c}+u_{IB,c}\right)}} \end{array}$$ (2j)

$$\begin{array}{ll} e^{-\beta \times 2\Delta G_{I,c}^{\text{free}}} & =\frac{\int_{\text{free}}dB\;\delta \left(r-r^{*}\right)\int dAe^{-\beta \left(U+u_{LA,c}+u_{LB,c}+u_{BA,c}+u_{BB,c}+u_{IA,c}+u_{IB,c}\right.)}}{\int_{\text{free}}dB\;\delta \left(r-r^{*}\right)\int dAe^{-\beta \left(U+u_{LA,c}+u_{LB,c}+u_{BA,c}+u_{BB,c}\right)}} \end{array}$$ (2k)

$$\begin{array}{ll} e^{-\beta \times 2\Delta G_{B,c}^{\text{free}}} & =\frac{\int_{\text{free}}dB\;\delta \left(r-r^{*}\right)\int dAe^{-\beta \left(U+u_{LA,c}+u_{LB,c}+u_{BA,c}+u_{BB,c}\right.)}}{\int_{\text{free}}dB\;\delta \left(r-r^{*}\right)\int dAe^{-\beta \left(U+u_{LA,c}+u_{LB,c}\right)}} \end{array}$$ (2l)

$$\begin{array}{ll} e^{-\beta \times 2\Delta G_{L,c}^{\text{free}}} & =\frac{\int_{\text{free}}dB\;\delta \left(r-r^{*}\right)\int dAe^{-\beta \left(U+u_{LA,c}+u_{LB,c}\right.)}}{\int_{\text{free}}dB\;\delta \left(r-r^{*}\right)\int dAe^{-\beta U}} \end{array}$$ (2m)

Note that the numerator of a given equation in Eq. 2 is generally the denominator of the previous equation, and Eqs 2k–2m contain contributions for both monomers, thus including a factor of 2. Eq. 2 may be written as a product of averages:

$$\begin{array}{cc} K_{eq} & ={\left\langle e^{-\beta u_{LA,c}}\right\rangle }_{\left(bound,U\right)}^{-1}{\left\langle e^{-\beta u_{LB,c}}\right\rangle }_{\left(bound,U+u_{LA,c}\right)}^{-1}{\left\langle e^{-\beta u_{BA,c}}\right\rangle }_{\left(bound,U+u_{L,c}\right)}^{-1}\\ & {\left\langle e^{-\beta u_{BB,c}}\right\rangle }_{\left(bound,U+u_{L,c}+u_{BA,c}\right)}^{-1}\\ & \times {\left\langle e^{-\beta u_{IA,c}}\right\rangle }_{\left(bound,U+u_{L,c}+u_{B,c}\right)}^{-1}{\left\langle e^{-\beta u_{IB,c}}\right\rangle }_{\left(bound,U+u_{L,c}+u_{B,c}+u_{IA,c}\right)}^{-1}\\ & {\left\langle e^{-\beta u_{o}}\right\rangle }_{\left(bound,U+u_{c}\right)}^{-1}{\left\langle e^{-\beta u_{a}}\right\rangle }_{\left(bound,U+u_{c}+u_{o}\right)}^{-1}\\ & \times {\left\langle e^{-\beta u_{o}}\right\rangle }_{\left(free,U+u_{c}\right)}{\left\langle e^{-\beta u_{I,c}}\right\rangle }_{\left(free,U+u_{L,c}+u_{B,c}\right)}{\left\langle e^{-\beta u_{B,c}}\right\rangle }_{\left(free,U+u_{L,c}\right)}\\ & {\left\langle e^{-\beta u_{L,c}}\right\rangle }_{\left(free,U\right)}\\ & \times \frac{c{}^{\circ}\int_{\text{bound}}dB\int dAe^{-\beta \left(U+u_{c}+u_{o}+u_{a}\right)}}{\int_{\text{free}}dB\delta \left(r_{AB}-r_{AB}^{*}\right)\int dAe^{-\beta \left(U+u_{c}+u_{o}\right)}}. \end{array}$$ (3)

In the above equation, we use the shorthand notation *u*_*L*,*c*_ = *u*_*LA*,*c*_ + *u*_*LB*,*c*_ (conformational restraint on the backbone atoms for loops 4 and 7 in both monomers A and B), *u*_*B*,*c*_ = *u*_*BA*,*c*_ + *u*_*BB*,*c*_ (conformational restraint on barrel backbone for both monomers), *u*_*I*,*c*_ = *u*_*IA*,*c*_ + *u*_*IB*,*c*_ (conformational restraint on interface residue sidechains for both monomers), and *u*_*c*_ = *u*_*L*,*c*_ + *u*_*B*,*c*_ + *u*_*I*,*c*_ (total conformational restraint). The last term written as a ratio of two integrals will be treated separately below.

Each average corresponds to a free energy change, for example, $e^{-\beta \Delta G_{LA,c}^{\text{bound}}}={\left\langle e^{-\beta u_{LA,c}}\right\rangle }_{\left(bound,U\right)}$ , where $G_{LA,c}^{\text{bound}}$ is the free energy change due to the addition of the restraining potential *u*_*LA*,*c*_ on the loops of chain A, in the bound state with no other restraints applied. Because *u*_*c*_ is intended to apply conformational restrictions that may be already partially restricted due to binding itself, the corresponding free energy contributions are expected to be smaller in the bound state than in the free state: $G_{c}^{\text{bound}}$ is thus expected to have smaller magnitude than $G_{c}^{\text{free}}$ . A similar expectation holds for the other applied potentials. The numbers in Table 1 do not always follow this expectation; however, we discuss this further below.

In terms of free energies, Eq. 3 can be written in the following form by pairing terms containing the same restraining potentials in the bound and free states:

$$\begin{array}{cc} K_{eq} & =\exp\left\{-\beta \left[\left(2\Delta G_{L,c}^{\text{free}}-\Delta G_{LA,c}^{\text{bound}}-\Delta G_{LB,c}^{\text{bound}}\right)+\left(2\Delta G_{B,c}^{\text{free}}-\Delta G_{BA,c}^{\text{bound}}-\right.\right.\right.\\ & \left.\Delta G_{BB,c}^{\text{bound}}\right)+\left(2\Delta G_{I,c}^{\text{free}}-\Delta G_{IA,c}^{\text{bound}}-\Delta G_{IB,c}^{\text{bound}}\right)+\left(\Delta G_{o}^{\text{free}}-\Delta G_{o}^{\text{bound}}\right)\\ & \left.\left.+\left(\Delta G_{\text{dist}+\text{a}}^{\text{restr}}-\Delta G_{a}^{\text{bound}}\right)\right]\right\} \end{array}$$ (4)

The 2nd to last terms in Eq. 4, defined as $e^{-\beta \Delta G_{\text{dist}+\text{a}}^{\text{restr}}}$ in Eq. 2i, can be written as

$$e^{-\beta \Delta G_{\text{dist}+\text{a}}^{\text{restr}}}\equiv c{}^{\circ}S^{*}I^{*}$$ (5)

where the term *S* ^∗^ addresses the removal of the relative angular restraints:

$$S^{*}=r^{*2}\int_{0}^{\pi }d\theta \;\sin\left(\theta \right)\int_{0}^{2\pi }d\phi \;e^{-\beta u_{a}}$$ (6)

and the term *I* ^∗^ can be recast as the difference in the potential of mean force (PMF) *W*(*r*) between the bound and free states, in the presence of the configurational, orientational, and axial restraints:

$$I^{*}=\int_{\text{bound}}\;\;\;dr_{AB}^{\prime }\;e^{-\beta \left[W\left(r_{AB}^{\prime }\right)-W\left(r_{AB}^{*}\right)\right]}\;.$$ (7)

In the above equations, *r*_*AB*_ is the scalar distance between the centers of masses of proteins *A* and *B* (*r* in Figure 1B), $r_{AB}^{*}$ is an arbitrary fixed location (*r* ^∗^, *θ* ^∗^, *ϕ* ^∗^) in the unbound region far from the other protein, *U* is the total potential energy of the system in the absence of restraints, potentials with lower case *u* are restraint potentials, and *W*(*r*) is the separation PMF in the presence of all restraints.

Several terms in Eq. 4 can be calculated analytically. For example, when protein **B** is in the unbound state sufficiently far away from its binding partner, the potential *U* + *u*_*c*_ is isotropic with respect to rotations about the angles Θ, Φ, and Ψ. This allows the term ${\left\langle e^{-\beta u_{o}}\right\rangle }_{\left(free,U+u_{c}\right)}$ in Eq. 4 to be calculated as

$$e^{-\beta G_{o}^{\text{free}}}=\frac{1}{8\pi^{2}}\int_{0}^{\pi }\sin\left(\Theta \right)d\Theta \int_{0}^{2\pi }d\Phi \int_{0}^{2\pi }d\Psi \;e^{-\beta u_{o}\left(\Theta ,\Phi ,\Psi \right)},$$ (8)

where *u*_*o*_ is a parabolic potential described by

$$u_{o}=\frac{1}{2}k_{\Theta }{\left(\Theta -\Theta_{0}\right)}^{2}+\frac{1}{2}k_{\Phi }{\left(\Phi -\Phi_{0}\right)}^{2}+\frac{1}{2}k_{\Psi }{\left(\Psi -\Psi_{0}\right)}^{2}.$$ (9)

Likewise, *S* ^∗^ in Eq. 6 can be calculated analytically, where *u*_*a*_ (*θ*, *ϕ*) in Eq. 6 is a parabolic potential given by

$$u_{a}=\frac{1}{2}k_{\theta }{\left(\theta -\theta_{0}\right)}^{2}+\frac{1}{2}k_{\phi }{\left(\phi -\phi_{0}\right)}^{2}$$ (10)

In Eqs 2a–2h, the numerators and denominators are partition functions before and after imposing restraints. Thus, the ratio of the partition function gives the free energy cost to impose the restraint. As shown schematically in Figure 2 (terms prior to separation), the restraints are imposed serially in the following order: *u*_*LA*,*c*_ → *u*_*LB*,*c*_ → *u*_*BA*,*c*_ → *u*_*BB*,*c*_ → *u*_*IA*,*c*_ → *u*_*IB*,*c*_ → *u*_*o*_ → *u*_*a*_.

The term in Eq. 2i includes both the free energy cost due to monomer separation in the presence of all the restraints (related to *I* ^∗^ in Eq. 7) and the free energy gain to release the axial angle restraints (related to *S* ^∗^ in Eq. 6). These terms combine to yield $\Delta G_{\text{dist}+\text{a}}^{\text{restr}}$ in Eq. 5 and Figure 2.

In Eqs 2j–2m, the numerators and denominators are partition functions before and after releasing restraints. Thus, the partition function ratio gives the free energy gain (lowering) upon release of the restraint. As shown schematically in Figure 2 (terms after monomer separation), the restraints are released serially in the reverse order of which they were applied: *u*_*o*_ → *u*_*IB*,*c*_ → *u*_*IA*,*c*_ → *u*_*BB*,*c*_ → *u*_*BA*,*c*_ → *u*_*LB*,*c*_ → *u*_*LA*,*c*_. The free energy change to release the restraints is gained for each monomer in the dimer. Thus, there is a coefficient of 2 in the exponents of Eqs 2k–2m. The exponent of Eq. 4 gives the binding free energy, written now in the order in which the terms are calculated:

$$\begin{array}{cc} \Delta G_{\text{bind}}= & -\Delta G_{LA,c}^{\text{bound}}-\Delta G_{LB,c}^{\text{bound}}-\Delta G_{BA,c}^{\text{bound}}-\Delta G_{BB,c}^{\text{bound}}-\Delta G_{IA,c}^{\text{bound}}-\Delta G_{IB,c}^{\text{bound}}\\ & -\Delta G_{o}^{\text{bound}}-\Delta G_{a}^{\text{bound}}+\Delta G_{\text{dist}+\text{a}}^{\text{restr}}+G_{o}^{\text{free}}+2\Delta G_{I,c}^{\text{free}}+2\Delta G_{B,c}^{\text{free}}\\ & +2\Delta G_{L,c}^{\text{free}} \end{array}$$ (11)

Each term in Eq. 11 is calculated by the free energy perturbation method. In other words, the potential of mean force (PMF) is calculated as a function of an order parameter, and the effects of imposing or releasing a given restraint are calculated by averaging the Boltzmann factor corresponding to that restraint over the unperturbed potential, as described further below.

### 2.2 Preparing Reference Structures

Reference structures are prepared as follows:

1) WT E,E (SS): We started from the E,Zn (SS) dimer of chains A and B in PDB structure 1HL4 (Strange et al., 2003), and we removed the Zn ion. The N-terminal acetyl-modification was also removed. Although eukaryotic SOD1 was N-terminally acetylated, the bacterial-expressed SOD1 used in most *in vitro* biophysical and structural studies was not acetylated (Stathopulos et al., 2003; Ahl et al., 2004; Arnesano et al., 2004; Lindberg et al., 2004; Vassall et al., 2006; Hörnberg et al., 2007; Svensson et al., 2010; Broom et al., 2015b).
2) WT E,E (SH): We started from chains A and B of PDB structure 2GBU (Hörnberg et al., 2007), a quadruple mutant C6A/C111A/C57A/C146A, which ablated the disulfide bond between C57 and C146. To recover the original WT primary sequence, we used Rosetta (Leaver-Fay et al., 2011) to perform mutations A6C, A111C, A57C, and A146C on 2GBU (without forming the disulfide bond), and then we relaxed the rotamer state of residues within 4.8Å of the four cysteines through the FastRelax mover (Khatib et al., 2011; Tyka et al., 2011; Gregorio Nivón et al., 2013; Conway et al., 2014).
3) A4V E,E (SS): We started from chains A and C of the crystal structure of A4V [PDB 6SPA (Chantadul et al., 2020)] and removed the Zn ions.
4) D101N E,E (SS): Because, to our knowledge, the structure of the D101N mutant had not yet been resolved, we prepared this reference structure starting from WT E,E (SS). We used Rosetta to perform the D101N mutation on the WT E,E (SS) structure. Then, we relaxed the rotamer state of residues within 4.8 Å of N101 through the FastRelax mover.
5) WT Cu,Zn(SS): We started from the holo WT reference structure, PDB 1HL5 (Strange et al., 2003).

For all four apo variants above, the histidine protonation states are 43HSP, 46HSD, 48HSD, 63HSE, 71HSE, 80HSE, 110HSD, and 120HSD, respectively, where HSD is protonated on the delta nitrogen, HSE is protonated on the epsilon nitrogen, and HSP is doubly protonated. These are the states observed in NMR structures [PDB 2AF2 (Banci et al., 2006) and 1L3N (Banci et al., 2002)]. For holo SOD1, the histidine protonation states are 43HSP, 46HSEM, 48HSDM, 63HSN, 71HSEM, 80HSEM, 110HSDM, and 120HSDM in which HSN, HSEM, and HSDM are histidine side chains that must be reparametrized from the putative CHARMM36m force field to facilitate metal binding (Section 2.3). The histidine protonation state is determined by the metal coordination in structure 1HL5 after building hydrogens using the GROMACS module pdb2gmx (Abraham et al., 2015). For all five variants, N- and C-termini are charged (NH3+ and COO−). The monomer reference structure for each SOD1 variant is taken as chain A of the respective dimer reference structure.

### 2.3 Reparametrized Histidines to Coordinate Ions

To model WT Cu,Zn (SS) SOD1, we reparametrized the coulomb partial charges of all histidines in coordination with metal ions, including histidines 46, 48, 63, 71, 80, 110, and 120. Histidine 63 bridges the Cu and Zn ions in the native structure and is doubly deprotonated (Banci et al., 2002). Such a residue is not present in the putative CHARMM force field. Reparametrized histidines have been used in previous studies for CHARMM27 (Peng et al., 2018) and AMBER and OPLSAA force fields (Wells et al., 2021) but not for the CHARMM36m force field used in this study. Histidines 46, 48, 120 interact with Cu, and histidines 71 and 80 interact with Zn. Reparametrizing these histidines corrects the partial charges due to the charge polarization in the electric fields of the metal ions and allows for proper metal-coordinating geometry consistent with experimental structures. The force field reparametrization is performed using a hybrid approach in which energy gradients (Maple et al., 1988; Waldher et al., 2010) and interaction energy with metal ions (Peng et al., 2018) are constrained to a target value. The partial charges of all atoms in the aromatic rings of the sidechains of metal-coordinating histidines are allowed to relax by minimizing the following loss function:

$$loss=w_{U}{\left(U-U{}^{\circ}\right)}^{2}+w_{g}\underset{\alpha ,i}{\sum }{\left(g_{\alpha ,i}-g_{\alpha ,i}{}^{\circ}\right)}^{2},$$ (12)

where *w*_*U*_ and *w*_*g*_ are weighting factors set to 1 and 10, respectively. *U*° is the quantum mechanical metal interaction energy of the subsystem consisting of the metal-coordinating histidine rings H46, H48, H63, H71, H80, and H120 and all atoms in the side chain of D83 in chain A of the holo SOD1 structure 1HL5. The quantum mechanical interaction energy was calculated in GAUSSIAN09 (Frisch et al., 2009) in a previous study (Peng et al., 2018). *g*_*α*,*i*_° is the potential energy gradient at the position of (or equivalently the force on) the Cu and Zn ions and should be zero in all directions *i* = 1, 2, 3 in the experimentally resolved structure. The subscript *α* = 1-4 includes the Cu and Zn ions in both chains A and H of structure 1HL5. As mentioned above, the calculation of interaction energy and gradient involves a subsystem in the vicinity of the metals. This includes Cu^2+^, Zn^2+^, all atoms in the histidine rings of the metal-coordinating amino acids (H46, H48, H63, H71, H80, and H120), and all atoms in the side chain of D83.

The partial charges in each reparametrized histidine are allowed to relax within a constrained range relative to the initial charge. The initial charges of H48, H110, and H120 are set to those in HSD in the CHARMM36m force field, and the initial charges of H46 and H71 are set to those of HSE in the CHARMM36m force field. The assignment of HSE or HSD is determined by metal coordination in 1HL5. The initial charge of the doubly deprotonated histidine H63 is designed from HSD in the CHARMM36m force field as follows: the hydrogen on the epsilon nitrogen (HE2) is removed, and then the surplus negative charge and the two previous nitrogen charges (ND1 and NE2) are redistributed evenly on the two nitrogens. Partial charges belonging to histidine 63 are restrained from being within ±0.5*e* of their initial charges, and the partial charges of the other atoms in the histidine side chains are constrained to be within ±0.1*e* of their respective initial charges.

Two additional constraints are also applied: 1) the charges of the nitrogens in H63 cannot be lower than −0.7, which is the partial charge of the deprotonated nitrogen in a neutral histidine, and 2) the partial charge of the CG atom in H63 must remain within ±0.1*e* of its initial charge. This latter constraint prevents the Zn ion from being shielded by the aromatic ring of H63, which prevents the SOD1 electrostatic loop from detaching from the beta barrel. This procedure is implemented in scipy constr-trust minimizer (Conn et al., 2000), and it gives the reproducible parameters in Table 2. With the reparametrized atomic partial charges in Table 2, the dimer and monomer of holo SOD1 remain in correct metal coordination for the full duration of our 200 ns MD simulations. From the last 160 ns of the monomer equilibrium trajectories, we calculated the all-atom root mean squared fluctuations (RMSF) for each amino acid coordinating either metal (H46, H48, H63, H71, H80, D83, and H120). Metalation structurally stabilized the coordinating amino acids, reducing the RMSF from $1.29\pm 1.28\overset{{}^{\circ}}{A}$ to $0.42\pm 0.17\overset{{}^{\circ}}{A}$ , a 67% decrease.

**TABLE 2:** Partial charges for reparametrized histidines in the CHARMM36m force field.

Atom	HSN	HSDM	HSEM
ND1	−0.944	−0.26	−0.8
HD1	—	0.42	—
CG	−0.15	0.05	0.12
CE1	0.75	0.15	0.15
HE1	0.5	0.23	0.03
NE2	−0.7	−0.8	−0.26
HE2	—	—	0.42
CD2	−0.156	0.12	0.05
HD2	−0.4	0.0	0.19

### 2.4 Equilibration

Before carrying out the potential of mean force (PMF) calculations, we obtained properly equilibrated initial structures as follows: three distinct systems required equilibration: bound dimer, interacting monomers during dimer separation, and isolated monomers. Special treatment was also applied to equilibrate the long disordered loops 4 and 7 of SOD1 for both dimer and monomer. All simulations were carried out using the CHARMM36m potential (Huang et al., 2017) in an explicit TIP3P solvent (Jorgensen et al., 1983), using GROMACS 2019.2 (Abraham et al., 2015) patched with PLUMED 2.5.2 (Bonomi, 2019) unless otherwise stated (e.g., Section 4). All simulations in this study were performed on the Sockeye computing cluster (UBC ARC Sockeye, 2019) using NVIDIA Tesla V100 GPU and Intel Xeon Silver 4216 CPU.

1) Dimer: A dodecahedron unit cell with box boundary 1.2 nm distance away from the closest atom on the protein was used for dimer simulations, wherein each variant was solvated with explicit TIP3P water, and K^+^ and CL^−^ ions were added to neutralize the system charge and maintain an aqueous salt concentration of 150 mM. System energy was then minimized through steepest descent until a maximum force < 100 kJ/mol/nm, followed by 300 ps NVT thermostat through the V-rescale method, with 1,000 kJ/mol/nm positional restraints on the heavy atoms. Protein and solvent thermostats had a coupling time of 0.1 ps. A time step of 2 fs was used in all simulations followed by a 300 ps NPT thermostat using the Parrinello−Rahman and V-rescale method with 1,000 kJ/mol/nm positional restraints on heavy atoms. The pressure coupling was isotropic with a coupling time of 2 ps and compressibility of 4.5 × 10^−5^ bar^−1^. Electrostatics was calculated by the PME method with order 4 and Fourier spacing of 0.16. The electrostatics cutoff and van der Waals cutoff were both 1.2 nm. LINCS constraints method of order 4 was applied on heavy atom-H bonds, with iteration set to 1. The temperature and pressure were maintained at 300 K and 1.0 bar, respectively.

Following the NPT thermostat, each dimer variant is relaxed for 200 ns using conventional MD, using the same method as the NPT thermostat, except that positional restraints are no longer applied. Because dimers are restrained progressively throughout the binding free energy calculation using the additional restraint potentials in Eq. 2, the PMF at different stages must be calculated with all the previous restraints present. As a result, equilibrated structures must be prepared with restraints successively applied. A 50 ns MD equilibration with the conformational restraint on loop backbone of chain A (*u*_*LA*,*c*_) was first implemented, followed by another 50 ns equilibration with both *u*_*LA*,*c*_ and the loop backbone restraint of chain B (*u*_*LB*,*c*_). In addition to the two loop backbone restraints, 10 ns MD equilibrations with the other restraints are then applied successively in the following order: *u*_*BA*,*c*_ → *u*_*BB*,*c*_ → *u*_*IA*,*c*_ → *u*_*IB*,*c*_ → *u*_Θ,*o*_ → *u*_Φ,*o*_ → *u*_Ψ,*o*_ → *u*_*θ*,*a*_ → *u*_*ϕ*,*a*_.

2) Interacting monomers during dimer separation: The initial protein structures used for constructing the separation PMF were taken from the final structure of the dimer equilibration simulation, with all restraints applied. The simulation unit cell was a dodecahedron with a box boundary of 3 nm from the closest atom on the protein to prevent the protein complex from interacting with its image during umbrella sampling on the separation distance. The procedure of solvation, ionization, energy minimization, and NVT/NPT equilibration followed the same procedure of the dimer. Following this, conventional MD with all restraints applied was run for 40 ns. The protein structures for chains A and B were then translated to various separation distances (Table 3) to be used as initial conditions for replica-exchange molecular dynamics umbrella sampling (REMD-US), as described in Section 2.5.
3) Monomer: Simulation box construction, solvation, ionization, energy minimization, and NVT/NPT equilibration followed the same procedure as the dimer above. Each apo monomer variant was then relaxed for 100 ns using conventional MD, and the holo monomer was relaxed for 200 ns using conventional MD. To prepare initial structures for PMF calculations in Eqs 2k–2m the above-equilibrated monomers were then successively restrained, starting with the loops using *u*_*L*,*c*_ (50 ns) and then the barrel using *u*_*B*,*c*_ (50 ns).
4) Dimer and monomer with disordered loops: The experimentally resolved apo SOD1 structures deposited on the protein databank generally have unresolved loops 4 and 7 (Elam et al., 2003a; Elam et al., 2003b; Strange et al., 2003; Roberts et al., 2007; Cao et al., 2008; Ahmad et al., 2009; Kevin et al., 2015), indicating these loops are disordered in both apo monomer and apo dimer. Sufficiently equilibrated structures with disordered loops thus have to be generated in order to properly seed the umbrella sampling simulations used in the PMF calculations (Section 2.5). This is done in two steps as follows.
For step 1, a reference structure with disordered loops is generated using reservoir replica-exchange molecular dynamics (R-REMD) simulation (Okur et al., 2007), using a modified version of GROMACS 4.6.7 (Hsueh and Plotkin). R-REMD simulation is only applied to the monomer structure of WT E,E (SS). The reservoir was generated by uniformly sampling 10,000 states from a 200 ns conventional MD simulation at 420 K. The multicanonical R-REMD simulation contained 40 replicas with temperatures ranging from 295 to 402.5 K and was run for 20 ns. The 300 K replica is clustered, and a representative structure from the largest cluster is extracted to proceed to the next step.
In step 2, for each variant, the monomer and each chain in the dimer have the positions of all the C_*α*_ atoms in loops 4 and 7 biased to the corresponding positions of the reference structure extracted from step 1. The spring constant and the target RMSD of the bias potential is 100 kcal/mol/Å^2^ and 0 Å. The biasing simulation is followed by either a 100 ns MD relaxation for monomers or a 40 ns MD relaxation for dimers.

**TABLE 3:** The parameters of REMD-US for calculating each free energy term. Units are ^*§*^ = kcal/mol/Å^2^ for Δ*G*_*LX*,*c*_ (*X* = *A*, *B* bound or free), Δ*G*_*BX*,*c*_, Δ*G*_*IX*,*c*_, and *W*(*r*). Units are ^*#*^ = kcal/mol/rad^2^ for $\Delta G_{\Theta ,o}^{\text{bound}}$ , $\Delta G_{\Phi ,o}^{\text{bound}}$ , $\Delta G_{\Psi ,o}^{\text{bound}}$ , $\Delta G_{\theta ,a}^{\text{bound}}$ , and $\Delta G_{\phi ,a}^{\text{bound}}$ .

SOD1 variant	Free energy term	Reaction coordinate range (Å or rad)	Number of umbrellas	Spring constant *k*	Length per umbrella (ns)
WT E,E (SS)	$\Delta G_{LA,c}^{\text{bound}}$	0.2–15.2	44	20 ^*§*^	220
	$\Delta G_{LB,c}^{\text{bound}}$	0.2–15.2	44	20	220
	$\Delta G_{BA,c}^{\text{bound}}$	0–1.4	8	20	20
	$\Delta G_{BB,c}^{\text{bound}}$	0–1.4	8	20	20
	$\Delta G_{IA,c}^{\text{bound}}$	0–2.0	11	10	20
	$\Delta G_{IB,c}^{\text{bound}}$	0–2.0	11	10	20
	$\Delta G_{\Theta ,o}^{\text{bound}}$	1.05–1.50	10	1,000 ^*#*^	20
	$\Delta G_{\Phi ,o}^{\text{bound}}$	1.60–2.00	9	1,000	20
	$\Delta G_{\Psi ,o}^{\text{bound}}$	−2.70 to − 2.20	11	1,000	20
	$\Delta G_{\theta ,a}^{\text{bound}}$	1.15–1.55	9	1,000	20
	$\Delta G_{\phi ,a}^{\text{bound}}$	1.50–1.95	10	1,000	20
	*W*(*r*)	22.9–39.2	35	10 (22.9–28.6 Å), 100 (27.5–30.8 Å), 10 (31.1–39.2 Å)	20
	$\Delta G_{I,c}^{\text{free}}$	0.1–4.5	23	10	20
	$\Delta G_{B,c}^{\text{free}}$	0–2.4	13	20	20
	$\Delta G_{L,c}^{\text{free}}$	0.2–15.2	44	20	220
	$\Delta G_{LA,c}^{\text{bound}}$	0.2–15.2	44	20	220
	$\Delta G_{LB,c}^{\text{bound}}$	0.2–15.2	44	20	220
WT E,E (SH)	$\Delta G_{BA,c}^{\text{bound}}$	0–1.4	8	20	20
	$\Delta G_{BB,c}^{\text{bound}}$	0–1.4	8	20	20
	$\Delta G_{IA,c}^{\text{bound}}$	0–2.0	11	10	20
	$\Delta G_{IB,c}^{\text{bound}}$	0–2.0	11	10	20
	$\Delta G_{\Theta ,o}^{\text{bound}}$	1.05–1.50	10	1,000	20
	$\Delta G_{\Phi ,o}^{\text{bound}}$	1.40–1.80	9	1,000	20
	$\Delta G_{\Psi ,o}^{\text{bound}}$	−2.80 to − 2.30	11	1,000	20
	$\Delta G_{\theta ,a}^{\text{bound}}$	1.15–1.55	9	1,000	20
	$\Delta G_{\phi ,a}^{\text{bound}}$	1.55–1.95	9	1,000	20
	*W*(*r*)	22.2–38.5	35	10 (22.2–26.1 Å), 100 (25.9–30.1 Å), 10 (30.4–38.5 Å)	20
	$\Delta G_{I,c}^{\text{free}}$	0.1–4.5	23	10	20
	$\Delta G_{B,c}^{\text{free}}$	0–2.4	13	20	20
	$\Delta G_{L,c}^{\text{free}}$	0.2–15.2	44	20	220
A4V E,E (SS)	$\Delta G_{LA,c}^{\text{bound}}$	0.2–15.2	44	20	300
	$\Delta G_{LB,c}^{\text{bound}}$	0.2–15.2	44	20	220
	$\Delta G_{BA,c}^{\text{bound}}$	0–1.4	8	20	20
	$\Delta G_{BB,c}^{\text{bound}}$	0–0.18	10	20	20
	$\Delta G_{IA,c}^{\text{bound}}$	0–2.0	11	10	20
	$\Delta G_{IB,c}^{\text{bound}}$	0–2.0	11	10	20
	$\Delta G_{\Theta ,o}^{\text{bound}}$	1.10–1.55	10	1,000	20
	$\Delta G_{\Phi ,o}^{\text{bound}}$	1.50–1.90	9	1,000	20
	$\Delta G_{\Psi ,o}^{\text{bound}}$	−2.80 to − 2.30	11	1,000	20
	$\Delta G_{\theta ,a}^{\text{bound}}$	1.15–1.55	9	1,000	20
	$\Delta G_{\phi ,a}^{\text{bound}}$	1.60–2.00	9	1,000	20
	*W*(*r*)	23.2–39.5	35	10 (23.2–27.7 Å), 100 (27.2–31.1 Å), 10 (31.4–39.5 Å)	20
	$\Delta G_{I,c}^{\text{free}}$	0.1–7.9	40	10	100
	$\Delta G_{B,c}^{\text{free}}$	0–3.0	16	50	100
	$\Delta G_{L,c}^{\text{free}}$	0.2–15.2	44	20	300
D101N E,E (SS)	$\Delta G_{LA,c}^{\text{bound}}$	0.2–15.2	44	20	220
	$\Delta G_{LB,c}^{\text{bound}}$	0.2–15.2	44	20	300
	$\Delta G_{BA,c}^{\text{bound}}$	0–2.0	11	20	20
	$\Delta G_{BB,c}^{\text{bound}}$	0–2.0	11	20	20
	$\Delta G_{IA,c}^{\text{bound}}$	0–2.0	11	10	20
	$\Delta G_{IB,c}^{\text{bound}}$	0–2.0	11	10	20
	$\Delta G_{\Theta ,o}^{\text{bound}}$	1.05–1.50	10	1,000	20
	$\Delta G_{\Phi ,o}^{\text{bound}}$	1.55–1.95	9	1,000	20
	$\Delta G_{\Psi ,o}^{\text{bound}}$	−2.80 to − 2.30	11	1,000	20
	$\Delta G_{\theta ,a}^{\text{bound}}$	1.15–1.55	9	1,000	20
	$\Delta G_{\phi ,a}^{\text{bound}}$	1.50–1.95	10	1,000	20
	*W*(*r*)	23.1–39.4	35	10 (23.1–28.8 Å), 100 (27.1–30.1 Å), 10 (30.4–39.4 Å)	20
	$\Delta G_{I,c}^{\text{free}}$	0–4.4	23	10	60
	$\Delta G_{B,c}^{\text{free}}$	0–2.4	13	50	60
	$\Delta G_{L,c}^{\text{free}}$	0.2–15.2	44	20	300
WT Cu,Zn (SS)	$\Delta G_{LA,c}^{\text{bound}}$	0.2–15.2	44	20	120
	$\Delta G_{LB,c}^{\text{bound}}$	0.2–15.2	44	20	120
	$\Delta G_{BA,c}^{\text{bound}}$	0–2.0	11	20	20
	$\Delta G_{BB,c}^{\text{bound}}$	0–2.0	11	20	20
	$\Delta G_{IA,c}^{\text{bound}}$	0–2.0	11	10	20
	$\Delta G_{IB,c}^{\text{bound}}$	0–2.0	11	10	20
	$\Delta G_{\Theta ,o}^{\text{bound}}$	1.05–1.50	10	1,000	20
	$\Delta G_{\Phi ,o}^{\text{bound}}$	1.60–2.05	10	1,000	20
	$\Delta G_{\Psi ,o}^{\text{bound}}$	−2.60 to − 2.20	9	1,000	20
	$\Delta G_{\theta ,a}^{\text{bound}}$	1.15–1.55	9	1,000	20
	$\Delta G_{\phi ,a}^{\text{bound}}$	1.55–1.95	9	1,000	20
	*W*(*r*)	22.9–39.2	36	10 (22.9–27.4 Å), 50 (26.9–27.5 Å), 100 (27.8–30.8 Å), 10 (31.1–39.2 Å)	20
	$\Delta G_{I,c}^{\text{free}}$	0–4.4	23	10	20
	$\Delta G_{B,c}^{\text{free}}$	0–2.4	13	10	20
	$\Delta G_{L,c}^{\text{free}}$	0.2–15.2	44	20	300

### 2.5 Potential of Mean Force Calculations

The calculations of all of the PMFs resulting from the applied restraints used replica-exchange MD combined with umbrella sampling (REMD-US). Distance, RMSD, or angle information obtained using PLUMED is analyzed, and a potential of mean force (PMF) for each reaction coordinate is obtained using the multistate Bennett acceptance ratio (MBAR) algorithm implemented through pymbar (Shirts and Chodera, 2008). After obtaining the PMF for a reaction coordinate, the cost of restraining that reaction coordinate can be computed using one of Eqs 2a–2m.

To implement REMD-US, a series of configurations along the reaction coordinate are generated by biased simulations. These configurations will serve as the starting configurations for the REMD-US windows. Starting from an equilibrated structure, a simulation with an increasing bias center and a simulation with a decreasing bias center are conducted in parallel. In other words, simulations are performed two at a time, with bias centers moving outwards from the original equilibrated structure. This ensures that the conformational changes across reaction coordinates are smooth and continuous.

Special treatment is applied to preparing the REMD-US initial configurations for the loop PMFs, to calculate $\Delta G_{LA,c}^{\text{bound}}$ , $\Delta G_{LB,c}^{\text{bound}}$ , and $G_{L,c}^{\text{bound}}$ . For loop terms, the two equilibrated structures (one with loops 4 and 7 structured and one with loops 4 and 7 disordered) generate four sets of biased simulations, using the above method of moving bias centers. The two biased simulations that start from the conformation with structured loops generate initial configurations with loop RMSD 0.2–7.0 Å, and the other two biased simulations that start from the conformation with unstructured loops generate initial configurations with loop RMSD 5.2–15.2 Å. The overlapped region, 5.2–7.0 Å, ensures sufficient exchange between umbrellas starting from different initial conformations.

The parameters in each REMD-US are listed in Table 3. Each window in REMD-US was initially run for 20 ns and was extended until the corresponding free energy contribution had converged. In other words, it did not change significantly as simulation time was increased (Supplementary Figure S7). Exchanges between neighboring windows are attempted every 1 ps and are accepted or rejected according to a Metropolis energy criterion.

Because the separation PMF *W*(*r*) has a steeper slope at the initiation of dissociation, higher umbrella spring constants are used for this region of rapidly changing PMF.

For all PMFs, the initial 50% of the REMD-US trajectory is discarded in constructing the PMF for calculating the free energy contribution.

### 2.6 Conformational, Orientational, and Angular Restraints

All the restraint potentials are harmonic. The same conformational restraints are applied to all four apo SOD1 variants, with parameters given in Table 4. The loop restraints of the holo SOD1 variant bias more closely to the native structure, with restraint center at 1.2 Å instead of 3.5 Å. Otherwise, the conformational restraint parameters are the same as apo ones. Although the conformational restraints for different variants take the same formula, they differ because the RMSD is calculated against different reference structures. The changes in PMFs after applying restraints are shown in Supplementary Figure S6.

**TABLE 4:** Conformational restraint parameters. The restraint parameters for WT Cu,Zn (SS) SOD1 are given inside parentheses when different from the apo parameters. Otherwise, they are the same.

Restraint	Reaction coordinate	Center	Spring constant *k*	Residues involved	Atoms
Potential		(Å)	(kcal/mol/Å^2^)		
*u*_*LA*,*c*_	Chain A loop backbone	3.5 (1.2)	10	49–83, 121–142	C, CA, N
*u*_*LB*,*c*_	Chain B loop backbone	3.5 (1.2)	10	49–83, 121–142	C, CA, N
*u*_*BA*,*c*_	Chain A barrel backbone	0.6	20	1–48, 84–120, 143–153	C, CA, N
*u*_*BB*,*c*_	Chain B barrel backbone	0.6	20	1–48, 84–120, 143–153	C, CA, N
*u*_*IA*,*c*_	Chain A interface sidechain	1.1	15	5, 7, 50–54, 114, 148, 150–153	All heavy
*u*_*IB*,*c*_	Chain B interface sidechain	1.1	15	5, 7, 50–54, 114, 148, 150–153	All heavy

The orientation restraint potentials confine the angles Θ, Φ, and Ψ to a variant-specific value, by three separate harmonic potentials *u*_Θ,*o*_, *u*_Φ,*o*_, and *u*_Ψ,*o*_ (Table 5). Likewise, the angular restraint harmonic potentials *u*_*θ*,*a*_ and *u*_*ϕ*,*a*_ restrain the angles *θ* and *ϕ* to values near those given in Table 5. Again, because the reference structure for each variant is different, each variant has a slightly different restraint center.

**TABLE 5:** Central angle values for orientational and angular restraints. The spring constants for the restraints are all 1,000 kcal/mol/rad^2^.

Restraint potential	Bias center (radians)				
	WT E,E (SS)	WT E,E (SH)	A4V E,E (SS)	D101N E,E (SS)	WT Cu,Zn (SS)
*u*_Θ,*o*_	1.37	1.39	1.32	1.32	1.35
*u*_Φ,*o*_	1.80	1.65	1.77	1.77	1.78
*u*_Ψ,*o*_	−2.38	−2.58	−2.43	−2.40	−2.45
*u*_*θ*,*a*_	1.31	1.36	1.31	1.30	1.35
*u*_*ϕ*,*a*_	1.76	1.71	1.78	1.79	1.77

Given the coordinate system used in Figure 1, another factor that weakly affects the final binding free energy is the choice of *r* ^∗^ in Eqs 6, 7, where *r* ^∗^ is determined as the last point in the separation PMF of each variant. The values used for *r* ^∗^ for the five variants (in the same order as in Table 5) are 38.8, 37.8, 38.8, 38.8, and 38.8 Å.

### 2.7 Total Simulation Time and Error Analysis

The accumulated simulation time in this work spent on each process includes equilibration (3.33* µs*), serial umbrella construction (2.12* µs*), REMD-US (175.46* µs*), and force field reparametrization (3.4* µs*). The total cumulative simulation time is thus 184.31* µs* divided into 111.01* μ*s of simulation time on the dimer system and 73.3* μ*s on the monomer system.

The binding free energy Δ*G*_bind_ error comes from the error propagation from each component term in Eq. 11. The error could come from two sources: the statistical error from the MBAR estimator (Shirts and Chodera, 2008) or the systematical error caused by insufficient convergence of REMD-US. In this study, the larger error of the two is used, so sufficient convergence of the REMD-US must be achieved to acquire a low enough Δ*G*_bind_ error. The detailed error calculation of each term is described in Supplementary Section S1.

## 3 Results

The dimer binding free energy of the five SOD1 variants described in the Methods section is calculated by the *ab initio* method detailed in Section 2.1, where a series of restraints are applied during separation to accelerate the convergence and then subsequently released. The free energy of imposing/releasing restraints is evaluated separately (see Figure 2) by applying the free energy perturbation method (Eqs 2a–2m on several potentials of mean force (PMFs) (Section 2.5). A tabulated list of free energy values contributing to the binding free energy is given in Table 1.

### 3.1 Free Energy of Monomer Separation

The largest free energy contribution is the cost of monomer separation $\left(\Delta G_{\text{dist}+\text{a}}^{\text{restr}}\right)$ . This term also contains a contribution from the relaxation of the axial angle restraints. $\Delta G_{\text{dist}+\text{a}}^{\text{restr}}$ is much larger than the experimental value of the binding free energy because of the numerous restraints that minimize unfavorable entropic factors in the binding process, which make sampling appreciably more efficient.

A4V E,E (SS) shows less binding free energy due to $\Delta G_{\text{dist}+\text{a}}^{\text{restr}}$ than WT E,E (SS). This is sensible because A4 is adjacent to the dimer interface, and its mutation, depending on the sidechain, could either remove dimer stabilizing interactions or stereochemically disrupt the dimer interface. WT E,E (SH) also has a smaller value of $\Delta G_{\text{dist}+\text{a}}^{\text{restr}}$ than that of WT E,E (SS). This is also sensible because residues 50–54 in loop 4 form part of the dimer interface. These are disordered in the disulfide-reduced state, as disulfide bond reduction removes the stable anchor between the loop to the *β* barrel.

Interestingly, although WT Cu,Zn(SS) SOD1 has more stable structure due to metal coordination and disulfide-bonded loop constraints, it has similar separation contribution $\Delta G_{\text{dist}+\text{a}}^{\text{restr}}$ to WT E,E (SS) and D101N E,E (SS) (Table 1). The separation contribution between monomers was similar in our calculations for all variants without mutations in the dimer interface due to the various restraints present in the calculation that minimize differences arising from entropic factors (Zhang et al., 2016).

All the REMD-US simulations converge within 20 ns per window (last row of Supplementary Figure S7). This fast convergence may be attributed to the convex binding interface for SOD1 dimers, in which no entangled loops are present, and the interface is mainly composed of *β*-sheets. A concave binding pocket, or a binding interface with entangled loops, often leads to artificially high unbinding free energies due to the long-time relaxations required for the structures on the dissociation pathway (Joshi and Lin, 2019; Walther Perthold and Oostenbrink, 2019).

### 3.2 Loop Contribution

**FIGURE 3:**
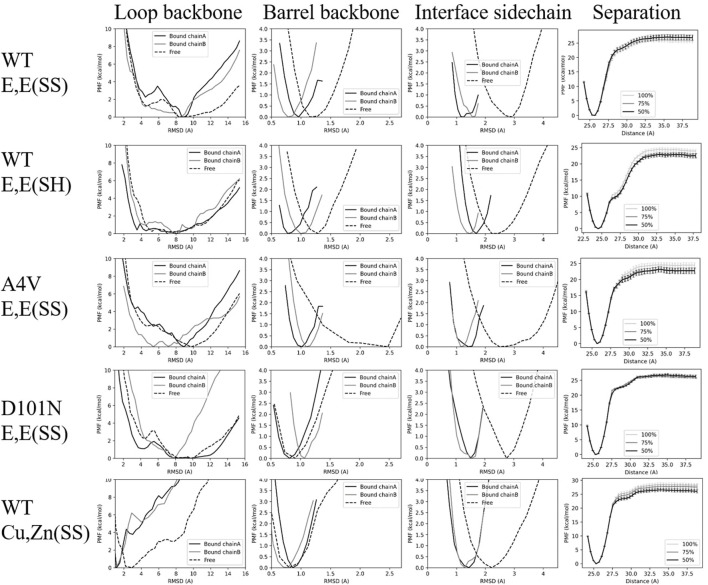
PMFs for various restraints for each variant. Each row shows the PMF surfaces for the various restraints applied to each given variant, and each column represents a given restraint: loop backbone, barrel backbone, interface sidechain, and inter-monomer separation distance. The PMFs labeled “Bound chain A/Bound chain B” correspond to varying umbrella restraints on the bound states of chain A or B respectively, while the PMFs labeled “free” correspond to varying umbrella restraints on the free state. The separation-distance PMFs are constructed using either the full, the last 75%, or the last 50% of the trajectories in REMD-US, as indicated in the legend.

We applied conformational restraints to the flexible loop region (loops 4 and 7) first because their relaxation time is otherwise very slow. We observed that several loop PMFs have a double-well structure (the first column of Figure 3; Supplementary Figure S6), in which the left well consists mainly of well-structured conformations and the right well consists mainly of entropically driven disordered structures. The double-well free energy surface suggests weak two-state-like transitions between ordered and disordered states of the long loops.

For WT E,E (SS) and A4V E,E (SS), the loop contributions from chain A are larger than chain B (i.e., $\Delta G_{LA,c}^{\text{bound}}>\Delta G_{LB,c}^{\text{bound}}$ , see Table 1), indicating that the folding of chain A facilitates the folding of chain B in the dimer. This cooperative folding effect is also reflected in the shape of PMF, where chain B has a deeper dip in the left well (RMSD ∼4Å ) than chain A. Moreover, the loop PMF for the free monomer has the widest and deepest free energy for the right well (RMSD ∼8Å ), suggesting that loop regions are further disordered in the free state.

For WT E,E (SH), restraining loops in the free monomer $\left(\Delta G_{L,c}^{\text{free}}\right)$ takes the largest energy among all the variants in this study, consistent with this variant’s lack of loop stabilization by the disulfide bond. Surprisingly however, its $\Delta G_{LA,c}^{\text{bound}}$ and $\Delta G_{LB,c}^{\text{bound}}$ are the lowest among the apo variants, suggesting that, in the apoprotein, the lack of disulfide bond may lower the free energy of stable structures or the disulfide bond may strain the apoprotein (but not the holoprotein). A similar effect has been observed by us previously (Das and Plotkin, 2013c) using simulated mechanical force spectroscopy probes. In this previous study, the formation of the disulfide bond in the apoprotein weakened the mechanical coupling between the disulfide bonding residues 57/146 and the rest of the protein. In contrast, for holo-SOD1, the presence of the disulfide bond mechanically stabilized those residues. One caveat in interpreting the results here is that the reference structure for WT E,E (SH) is already less compact than that of the other disulfide-bonded variants (Hörnberg et al., 2007), making a direct comparison of the free energy cost to restrain loops less straightforward.

For D101N E,E (SS) mutant SOD1, the free energy cost to restrain the loops on chain B is much larger than the free energy cost to restraint loop on chain A (Table 1), which indicates that this mutation induces anticooperativity in the folding of loops, as opposed to apo WT and apo A4V. The loss of cooperative folding due to this mutation is discussed further in Section 4.1.

For WT Cu,Zn(SS), the loops are greatly stabilized by the metal cations, so a loop conformational restraint with a smaller bias center, 1.2 Å, is imposed (see bottom-left subpanel in Supplementary Figure S6). Despite this tighter restraint, both dimer and monomer loop free energy contributions were the smallest among the variants studied. The shift in free energy surface upon metalation for SOD1 towards a more structured free energy minimum (Figure 3, lower left panel) is consistent with previous experimental results showing that the presence of Zn facilitated the folding of disordered loops (Kayatekin et al., 2008).

The net free energy contribution from loops to the binding free energy is given by $2\Delta G_{L,c}^{\text{free}}-\Delta G_{LA,c}^{\text{bound}}-\Delta G_{LB,c}^{\text{bound}}$ (Table 6). Each Δ in the equation is the cost to constrain the loops, so a smaller constraining cost in the dimer means a relatively larger free energy decrease due to conformational relaxation in the monomer *versus* the dimer. We thus found that loop free energy has a destabilizing effect upon dimerization for all variants studied except for WT E,E (SS). Consistent with previous experimental results (Hörnberg et al., 2007), the loop stability penalty is the largest for the disulfide-reduced variant WT E,E (SH).

**TABLE 6:** Grouping of values in Table 1 in different combinations. Top: the net free energy change of different conformational freedoms upon monomerization. This grouping is used in Eq. 4. Middle 4 rows: the conformational free energy contributions to dimer and monomers. The free energy changes ΔΔ*G* compared with WT E,E (SS) are also calculated. Bottom 4 rows: the dimer binding free energies excluding the contributions from loops (row 1), excluding loops and barrel (row 2), excluding loops, barrel, and interface (row 3), and excluding barrel only (after constrained loops; row 4).

Net free energy change upon monomerization	WT E,E (SS) PMF (kcal/mol)	WT E,E (SH) PMF (kcal/mol)	A4V E,E (SS) PMF (kcal/mol)	D101N E,E (SS) PMF (kcal/mol)	WT Cu,Zn (SS) PMF (kcal/mol)
Loop backbone $2\Delta G_{L,c}^{\text{free}}-\Delta G_{LA,c}^{\text{bound}}-\Delta G_{LB,c}^{\text{bound}}$	−0.07 ± 3.38	4.73 ± 0.75	1.46 ± 1.64	1.58 ± 1.22	6.91 ± 2.65
Barrel backbone $2\Delta G_{B,c}^{\text{free}}-\Delta G_{BA,c}^{\text{bound}}-\Delta G_{BB,c}^{\text{bound}}$	3.83 ± 0.22	3.60 ± 0.13	5.99 ± 0.53	−1.04 ± 0.06	−0.00 ± 0.10
Interface sidechain $2\Delta G_{I,c}^{\text{free}}-\Delta G_{IA,c}^{\text{bound}}-\Delta G_{IB,c}^{\text{bound}}$	11.18 ± 0.30	6.38 ± 0.09	8.13 ± 0.14	10.42 ± 0.85	4.80 ± 0.15
Orientational angles $G_{o}^{\text{free}}-\sum_{X=\Theta ,\Phi ,\Psi }\Delta G_{X,o}^{\text{bound}}$	4.87 ± 7.49	5.14 ± 7.57	5.65 ± 7.57	4.99 ± 7.51	5.62 ± 7.59
All conformational freedom $\sum_{X=L,B,I}\left(2\Delta G_{X,c}^{\text{free}}-\Delta G_{XA,c}^{\text{bound}}-\Delta G_{XB,c}^{\text{bound}}\right)$	14.94 ± 6.41	14.71 ± 3.91	15.58 ± 6.12	10.96 ± 5.72	11.70 ± 2.85
Free energy cost to restrain conformation in monomers/dimer					
$\sum_{X=L,B,I}2\Delta G_{X,c}^{\text{free}}$	25.53 ± 2.80	23.16 ± 0.49	27.02 ± 1.42	21.98 ± 0.99	13.94 ± 2.65
$\sum_{X=L,B,I}\left(\Delta G_{XA,c}^{\text{bound}}+\Delta G_{XB,c}^{\text{bound}}\right)$	10.59 ± 1.93	8.45 ± 0.59	11.44 ± 0.97	11.02 ± 1.12	2.23 ± 0.07
$\Delta \;\left(\sum_{X=L,B,I}2\Delta G_{X,c}^{\text{free}}\right)$	—	−2.37 ± 2.84	1.48 ± 3.14	−3.55 ± 2.97	−11.60 ± 3.86
$\Delta \left(\sum_{X=L,B,I}\left(\Delta G_{XA,c}^{\text{bound}}+\Delta G_{XB,c}^{\text{bound}}\right)\right)$	—	−2.14 ± 2.02	0.84 ± 2.16	0.43 ± 2.23	−8.36 ± 1.93
Δ*G*_bind_ excluding the certain conformational contribution					
$\Delta G_{\text{bind}}^{no\;L}$	−3.38 ± 0.87	−3.69 ± 0.65	0.80 ± 0.98	−8.32 ± 0.81	−11.88 ± 1.59
$\Delta G_{\text{bind}}^{no\;L,B}$	−7.21 ± 0.85	−7.29 ± 0.64	−5.19 ± 0.91	−7.28 ± 0.81	−11.88 ± 1.59
$\Delta G_{\text{bind}}^{no\;L,B,I}$	−18.39 ± 0.82	−13.67 ± 0.63	−13.32 ± 0.90	−17.70 ± 0.52	−16.67 ± 1.58
$\Delta G_{\text{bind}}^{no\;B}$	−7.28 ± 2.88	−2.56 ± 0.93	−3.73 ± 1.62	−5.70 ± 1.42	−4.97 ± 2.45

Perhaps surprisingly, for WT E,E (SS), the loop free energy does not disfavor dimerization in our calculation, and dimerization has almost no effect on loop stability. Moreover, the loops do not appear to have reduced conformational freedom in the dimer. The conformational fluctuations of the loops in the dimer have an average RMSF of 3.59 Å, while the monomers have a slightly smaller average RMSF of 3.26 Å. Rather than supporting a mechanism of entropy-enthalpy compensation upon dimerization acting on loop conformations, this supports significant conformational freedom of the loops in the WT E,E (SS) dimer.

### 3.3 *β* − Barrel Contribution

The *β*-barrel backbone is the next structural region restrained after the loops, before monomer separation. By comparing the free energy cost to restrain the barrel backbone in monomers *versus* dimer ( $2\Delta G_{B,c}^{\text{free}}-\Delta G_{BA,c}^{\text{bound}}-\Delta G_{BB,c}^{\text{bound}}$ , Table 6), we sensibly found that the barrel backbone generally had larger flexibility in unbound the monomer than in the bound dimer and thus opposed dimerization. Interestingly, the magnitude of this effect was often as large as that of the loops.

D101N E,E (SS) is again exceptional in having a rigid barrel backbone in the unbound monomer, which approaches the stability of the WT Cu,Zn(SS) *β*-barrel (Table 1). The barrel in the monomer is actually more stable than in the dimer, indicating that the *β*-barrel conformational free energy favors rather than opposes dimer binding.

A4V E,E (SS) has the least stable *β*-barrel of the variants in this study (Table 1), and the backbone conformational free energy of A4V E,E (SS) destabilizes the dimer and opposes its formation most strongly of all the variants.

To ensure that the above effects on D101N E,E (SS) and A4V E,E (SS) were not artifacts of insufficient sampling, we used a larger spring constant, *k* = 50 kcal/mol/Å^2^, and longer simulation time for the REMD-US method for constructing the PMF for $\Delta G_{B,c}^{\text{free}}$ (Table 3; Supplementary Figure S7).

### 3.4 Interface Contribution

The final conformational restraint is applied to all (sidechain and backbone) heavy atoms of the dimer binding interface residues. We found the sensible result that the bound states always had increased interface stability relative to the free state (the third column in Figure 3 and $2\Delta G_{I,c}^{\text{free}}-\Delta G_{IA,c}^{\text{bound}}-\Delta G_{IB,c}^{\text{bound}}$ in Table 6), meaning that ordering of the interface sidechains strongly opposes dimer binding. This effect is largely entropic, as the energetic terms mediated by these sidechains (as well as other atoms) that favor dimer binding are accounted for in the PMF calculation for monomer separation (Section 3.1). The enhanced structural order of loops upon metalation also reduces the conformational disorder of interfacial residues as roughly five interface residues reside in loop 4.

### 3.5 Orientational and Angular Contribution

The remaining PMFs for orientational and angular restraints in bound states all converge rapidly, with almost perfect overlap between PMFs constructed using either the last 75% or last 50% of the sampling trajectories (Supplementary Figures S1–S5). As expected, all the orientational free energy contributions in the bound state are negligibly small (Table 1). On the contrary, their contributions in the free monomer state, determined analytically and similar for all, are significant. In the free monomer state, the orientational restraints cost 7.62–7.63 kcal/mol. The opposition to dimer binding by restriction of rotational freedom of independent monomers is simple universal free energy cost that is variant and independent.

Because the equilibrium constant in Eq. 1 has dimensions of volume, the calculation involves a volume that is contained in terms *S* ^∗^ and *I* ^∗^ (Eqs 6, 7). The angular restraints are manifested in *S* ^∗^, representing the area available to chain B on the sphere of $r^{*}\approx \left(37.8-38.8\right)\overset{{}^{\circ}}{A}$ surrounding chain A, and are $\approx \left(5.2-5.5\right){\overset{{}^{\circ}}{A}}^{2}$ for all the SOD1 variants. In other words, the solid angle is restrained to be $∼\frac{1}{270}$ of the entire 4*π* during the separation.

### 3.6 Comparison of Δ*G*_bind_ With Experiment

The binding free energies calculated in this study (Table 1) are systematically weaker than the experimentally determined values, which is an issue reported before (Siebenmorgen and Zacharias, 2019) for the method we have used here. It may be rooted in inaccuracies of the non-polarizable force field we have used here for molecular dynamics simulations (CHARMM36m), particularly when used to evaluate protein binding free energies (Hazel et al., 2018). The mechanically induced unfolding of SOD1 has been observed to have a mechanism that is robust to force field and coarse-grained model for early events but sensitive to the force field and model for late stage unfolding events when the protein is more significantly disordered (Habibi et al., 2016). Differences in binding free energy between SOD1 variants (i.e., ΔΔ*G*_bind_) may be more robust to the force field, and we compare these here as well.

The dimer binding free energy Δ*G*_bind_ of WT E,E (SS) has been experimentally measured by several research groups. Published values include −12 kcal/mol^52^, −11.0 ± 0.4 kcal/mol at 23°C (Broom et al., 2015b), and −10.3 ± 0.5 kcal/mol at 37°C (Broom et al., 2015b). As mentioned above, this is much larger in magnitude than our calculated value of ≈−3.5 kcal/mol.

The conformational entropy increase associated with disulfide reduction for WT E,E (SH) was reported to lead to the dissociation of the apo SOD1 dimer in physiological concentration (Lindberg et al., 2004) and has also been reported to have at least 4 orders of decrease in the association constant (Arnesano et al., 2004), corresponding to about a 5.5 kcal/mol shift towards weaker Δ*G*_bind_. In our calculation, the ΔΔ*G*_bind_ between WT E,E (SH) and WT E,E (SS) is 4.5 ± 3, which is quite close to this experimental value. The dissociation constant for WT E,E (SH) has been reported to be approximately 85 ± 50 mM based on measured transient populations (Sekhar et al., 2015), which correspond to the binding free energy between −1 kcal/mol and −2 kcal/mol. While these experiments have measured marginal stability for the E,E (SH) dimer, our calculations have yielded a marginal instability for the E,E (SH) dimer of +1.04 ± 0.94 kcal/mol.

The experimental binding free energy Δ*G*_bind_ for A4V E,E (SS) SOD1 has been reported as − 7.2 ± 0.2 at 23°C (Broom et al., 2015b), −7.9 ± 0.7 at 25°C (Broom et al., 2015b), and −6.4 ± 0.3 kcal/mol at 37°C (Broom et al., 2015b). It has also been reported that the dissociation constant (*K*_*d*_) is in the mM range (corresponding to *G*_bind_ ≈ − 4 kcal/mol) (Hörnberg et al., 2007). The ΔΔ*G*_bind_ of A4V E,E (SS) relative to WT E,E (SS) based on these experiments is 3.9 ± 0.6 kcal/mol at 37°C or 3.8 ± 0.5 kcal/mol at 23°C. In our calculations, the ΔΔ*G*_bind_ of A4V E,E (SS) relative to WT E,E (SS) is about 5.7 ± 3.3, which is higher than the value from these experiments but still in the approximate experimental range.

The collective increase in the loop, barrel, and interface conformational free energy upon monomerization for all variants studied (Table 6) is consistent with experimental observations of extensive disruption of native structure upon apo SOD1 dimer dissociation (Broom et al., 2015b). These experimental measurements are based on the overall heat capacity and enthalpy changes upon dissociation, so they are not structurally resolved.

We may compare the total conformational free energy change between a variant and WT in both the free monomer and bound dimer states (Table 6). This calculation shows that the D101N mutation on the apoprotein [comparing to WT E,E (SS)] has a stabilizing effect on the free monomer $\left(\Delta \left(\sum_{X=L,B,I}2\Delta G_{X,c}^{\text{free}}\right)=-3.55\pm 2.97\right)$ , and almost no effect in the bound dimer $\Delta \left(\sum_{X=L,B,I}\left(\Delta G_{XA,c}^{\text{bound}}+\Delta G_{XB,c}^{\text{bound}}\right)\right)=0.43\pm 2.23$ ).

The thermodynamic effects of D101N have been experimentally resolved for the unfolding of the apo monomer (ΔΔ*G*_D-M_ = −0.80 kcal/mol) and unfolding of the apo dimer $\Delta \Delta G_{{\text{D}-\text{M}}_{2}}=-0.75$ kcal/mol) (Byström et al., 2010). However, these numbers couple in the unfolding free energy and rely on a linear extrapolation from 5.8 M urea to 0 M urea (Byström et al., 2010), which does not permit a direct comparison to our values for the dimer binding free energy. Under the additional assumption that the dimer association rate is the same for WT and D101N, the experimental value of the difference in dimer binding free energy mutant to WT is $-2.3RT\log_{10}\left(k_{d}^{WT}/k_{d}^{mut}\right)$ giving a value of ΔΔ*G* ≈ 0.01 kcal/mol for D101N, or essentially equal to the WT dimer binding free energy.

The difference in the binding free energy of D101N-WT heterodimer to the WT and mutant homodimers, Δ*G*_het_ = 2Δ*G*_WT-mut_ − Δ*G*_WT-WT_ − Δ*G*_mut-mut_, has also been experimentally resolved by Shi et al. (2016) to be −0.71 kcal/mol. Based on our dimer binding free energies, this gives a predicted value for the heterodimer binding free energy of −5.4 kcal/mol for the D101N-WT apo heterodimer.

Our calculations also show that the A4V mutation conformationally destabilizes both bound dimer ( $\Delta \left(\sum_{X=L,B,I}\left(\Delta G_{XA,c}^{\text{bound}}+\Delta G_{XB,c}^{\text{bound}}\right)\right)=0.84\pm 2.16$ kcal/mol) and free monomer ( $\Delta \left(\sum_{X=L,B,I}2\Delta G_{X,c}^{\text{free}}\right)=1.48\pm 3.14$ kcal/mol). Because the free monomer has larger required constraining free energy than the bound dimer, this conformational disruption further opposes dimer binding. To our knowledge, there is no direct experimental measurement of these free energies. However, it has been shown that A4V is one of the most destabilizing mutants for both monomer and dimer unfolding (ΔΔ*G*_D-M_ = 1.62 kcal/mol and $\Delta \Delta G_{{2\text{D}-\text{M}}_{2}}=4.31$ kcal/mol) (Lindberg et al., 2005).

In our calculations, the ΔΔ*G*_bind_ between D101N E,E (SS) and WT E,E (SS) is ≈−3.29±3.22 kcal/mol, which is a substantially increased dimer binding affinity for D101N E,E (SS). This was largely due to its less flexible barrel backbone for the free monomer—the D101N mutation is located in the *β*-barrel. Byström et al. reported an increase in the dimer stability for the ALS mutant E,E (SS) D101N of 0.75 kcal/mol^8^, which is more modest than the number we observe but is a stabilizing mutation. Such mutants are important in understanding the sources of pathology in ALS, which can evidently arise from additional factors other than the loss of native state stability.

To our knowledge, the dimer binding free energy of WT Cu,Zn (SS) has not yet been reported experimentally. In our calculations, we were somewhat surprised to see that WT Cu,Zn (SS) showed only modestly higher binding affinity than WT E,E (SS), by about ΔΔ*G*_bind_ ≈ − 1.5 ± 3.8. We suspect this is an underestimate, which, in any event, future experiments may be able to test. This increased binding affinity for holo SOD1 arises from increased conformational stability in the free monomer for all regions considered here—loop, barrel, and interface (see Table 6)—indicating that metalation of SOD1 conformationally stabilizes the free monomer.

### 3.7 Disulfide Reduction Mainly Affects the Loop Contribution to Dimer Stability: D101N Mutation Mainly Affects the Loop and Barrel Backbone Contribution to Dimer Stability

Because the binding free energy is calculated in a modular fashion, we can dissect the binding free energy by excluding certain free energy contributions. As a specific example, the binding free energy excluding the loop contribution would be $\Delta G_{\text{bind}}^{no\;L}=-\Delta G_{BA,c}^{\text{bound}}-\Delta G_{BB,c}^{\text{bound}}-\Delta G_{IA,c}^{\text{bound}}-\Delta G_{IB,c}^{\text{bound}}-\Delta G_{o}^{\text{bound}}-\Delta G_{a}^{\text{bound}}+\Delta G_{\text{dist}+\text{a}}^{\text{restr}}+\Delta G_{o}^{\text{free}}+2\Delta G_{I,c}^{\text{free}}+2\Delta G_{B,c}^{\text{free}}$ (Table 6). The free energy excluding the loop energy terms for $\left(\Delta G_{\text{bind}}^{no\;L}\right)$ for the WT E,E (SS) and WT E,E (SH) variants is −3.38 ± 0.87 and −3.69 ± 0.65 kcal/mol, respectively, which are in mutual agreement within the error bars. Thus the difference of the binding free energy due to the reduction of the disulfide bond is mainly reflected by the loop contribution.

Likewise, if we ablate both the loop and barrel backbone contribution $\left(\Delta G_{\text{bind}}^{no\;L,B}\right)$ , the free energy for WT E,E (SS), WT E,E (SH), and D101N E,E (SS) would be −7.21 ± 0.85, −7.29 ± 0.64, and −7.28 ± 0.81, respectively, which mutually agree within the error bars. This suggests that D101N mutation mainly affects the loop and barrel backbone contribution.

## 4 Discussion

### 4.1 Variants That Affect Loops 4 and 7 Disrupt the Cooperative Folding of Loops in the Dimer

Conformational restraints in the bound dimer state are imposed first on chain A and then on chain B, so one might expect a smaller free energy cost to constrain chain B than for chain A (positive cooperativity). For loop constraints, this positive cooperative folding effect (i.e., $\Delta G_{LA,c}^{\text{bound}}>\Delta G_{LB,c}^{\text{bound}}$ ) is only observed for the A4V E,E (SS) (Table 1), with a modest but not statistically significant positive cooperativity for WT E,E (SS). The holo SOD1 protein has relatively small $\Delta G_{LA,c}^{\text{bound}}$ and $\Delta G_{LB,c}^{\text{bound}}$ , but the anticooperativity is statistically significant. The WT E,E (SH) and D101N E,E (SS) variants significantly disrupt cooperative loop folding. Both disulfide reduction and D101N mutations are either within the loops or close to the loop regions, so the anti-cooperative effect may be due to the loop structures having conformational ensembles that are modified by mutation or disulfide reduction. Previous studies have shown that mutation could affect cooperative folding (Batey et al., 2005; Rogers, 2020) and that disordered structures (such as disordered loops) could affect long-range ( > 10 nm) cooperative folding (Gruszka et al., 2016). In the disulfide-reduced and D101N variants, well-structured loops in chain A may strain the native structure of chain B so that loop disorder is enhanced for chain B in the context of the dimer.

Similarly, we notice from Table 1 that positive cooperativity of the barrel is present for the WT holo and apo variants but is lost for all mutants, as well as the disulfide-reduced variant. Positive cooperativity of the interface sidechains is present for all variants, except unexpectedly for the WT apoprotein. One caveat to this analysis is that the free energies due to adding constraints are implemented in a specific order. We have not pursued alternate orderings of adding the constraints here.

### 4.2 Dissociation of Dimer Is Not Sufficient to Explain ALS Pathogenesis or Progression

Although SOD1 dimer dissociation has been thought to be an initial event in ALS pathogenesis, the result that D101N increases dimer binding affinity supports a view that properties other than native stability contribute to ALS-associated cellular toxicity (Rodriguez et al., 2005). These properties may include decreased initial folding rate from nascent protein, thus increasing the probability of off-pathway misfolding and aggregation (Bruns and Kopito, 2007), enhanced aggregation propensity due to reduction of repulsive negative charge (Sandelin et al., 2007), or increased tendency to form heterodimers with WT SOD1 (Shi et al., 2016).

### 4.3 The Validity of the Δ*G*_bind_ Calculation

Our calculations have fairly large error bars, and, in some cases, [WT E,E (SS) SOD1] appeared to yield smaller values than those determined experimentally. Hansen and WilfredGunsteren (2014) described three essential components of a reliable free energy calculation: an adequate estimator, a suitable model Hamiltonian, and sufficient sampling. For the free energy estimator we have used here, MBAR, seen as a binless extension of the WHAM method, has been shown to be accurate in several studies (Fajer et al., 2009; Tan et al., 2012). Furthermore, errors due to differences in the free energy estimator have been shown to be less significant than insufficient sampling (Christ and Fox, 2014). The other two components, suitable Hamiltonian and sufficient sampling, are discussed further below.

A long-standing concern of free energy calculations is the accuracy of the force field in quantifying biomolecular processes such as protein folding and binding (Gathiaka et al., 2016). Although the protein force fields have improved over time (Piana et al., 2014), protein conformational changes, including folding and binding, are still difficult to accurately describe by classical force fields (Best and Mittal, 2010; Piana et al., 2011; Lindorff-Larsen et al., 2012; Piana et al., 2014; Hazel et al., 2018). Even for small molecules, the free energy costs to restrain conformations upon binding to proteins have shown large variations from different force fields (Lahey and Rowley, 2020). For a suitable model Hamiltonian, CHARMM36m was chosen due to its accurate parameterization of both ordered and disordered structures (Huang et al., 2017), as well as its accuracy in unfolding free energy calculations (Lee and Kuczera, 2021), which here should accurately account for the free energy of restraining the disordered loops in SOD1.

In our calculations, sufficient sampling required at least three elements: a valid initial structure, convergence of the PMF, and proper seeding configurations in REMD-US. These are detailed further below.

1) Valid initial structure: The calculation method developed by Roux and co-workers that we use here (Woo and Roux, 2005; Gumbart et al., 2013a) has been shown to give higher binding affinities when starting from an experimentally determined protein complex than from docked complexes (Siebenmorgen and Zacharias, 2019). A reliable protein complex structure is thus essential for an accurate binding free energy calculation (Siebenmorgen and Zacharias, 2019). Experimental NMR structures of obligate apo monomers (PDB 1RK7) have been determined (Banci et al., 2003) and utilized in previous computational studies of misfolding-specific epitope prediction (Peng et al., 2018) and forced unfolding (Habibi et al., 2017; Habibi et al., 2018). However, we required an E,E (SS) SOD1 dimer structure in this study, which has not yet been experimentally resolved to our knowledge. The E,E (SS) reference structures used in this study are thus modified from RCSB holo or partially metallated structures (Section 2.2), wherein the modifications involved the removal of ions and the required mutations to the variants considered here. The WT E,E (SH) SOD1 calculation used the experimentally resolved E,E (SH) dimer structure (Hörnberg et al., 2007). The rotamer states of neighboring residues of the mutation sites were relaxed using Rosetta (Leaver-Fay et al., 2011) to accelerate the equilibration of sidechain packing. This was followed by 100–200 ns of equilibrium MD to ensure the configuration had relaxed to the lowest free energy state (Section 2.4).
2) PMF convergence: Because multiple PMFs are involved in the calculation of Δ*G*_bind_, the numerical answer is susceptible to accumulation of errors (Section 2.7). Convergence of the PMFs is thus essential to assure the accuracy of the result. To assess the convergence of the PMFs, each free energy contribution is calculated with accumulated REMD-US trajectories (Supplementary Figure S7). REMD-US is extended until each energy contribution is stable and does not change significantly (Supplementary Figure S7). Most of the free energy contributions reached stable values within 20 ns per umbrella. However, the loop free energy terms required much longer time to reach stable values. Opposed to other studies where most of the computing resources were spent on the positional separation $\left(\Delta G_{\text{dist}+\text{a}}^{\text{restr}}\right)$ (Woo and Roux, 2005; Gumbart et al., 2013a), in this study, constructing loop PMF consumed most of the computational resources. For example, the total REMD-US trajectory length for calculating WT E,E (SS) $\Delta G_{LA,c}^{\text{bound}}$ was over 13-fold higher than that used for *W*(*r*) (9,680 vs. 700 ns). One reason for this difficulty was the high entropy in the large RMSD regime, which required a large phase space to be sampled before equilibrium could be achieved. RMSD may not be the optimal reaction coordinate to calculate the PMF (Fajardo and Heyden, 2021), and other reaction coordinates optimized for constructing conformational landscapes may be applied in future studies (Ahalawat and Mondal, 2018). Other advanced simulation methods such as two-dimensional REMD-US (Gee and Scott Shell, 2011) may also be used to accelerate the convergence.
3) REMD-US seeding configurations: The PMFs associated with loops often had a double-well topography. In practice, their convergence was strongly affected by the initial seeding configuration in each REMD-US window. As described in Section 2.5, the initial configurations in each REMD-US window simulation were RMSD-steered starting from one of two equilibrated structures: a structure at RMSD ∼ 4Å in the enthalpically driven minimum of the free energy *versus* RMSD or a structure at RMSD ∼ 8Å in the entropically driven minimum. We found that constructing the PMFs using umbrella sampling with initial configurations steered solely from one of the equilibrated ensembles resulted in PMFs that were significantly different and thus nonconverged. Specifically, the umbrella sampling did not find stable structures that were not initially seeded. Thus, the resulting PMFs are missing one of the free energy wells (Supplementary Figure S8). As a result, we seeded our umbrella sampling conformations from both the small and large RMSD basins. In the barrier region of the PMF, RMSD = 5.2 – 7.0Å, we simply took an even mixture of initial conformations. Thus, there was twice the umbrella density there than in other regions of the PMF. A possible future direction to reduce the number of umbrellas and/or increase the accuracy of umbrella sampling in the free energy barrier region could be to use structural interpolation methods such as FRODAN (Farrell et al., 2010) or NMSim (Ahmed et al., 2011) to generate more representative seeding conformations in the transition region.

### 4.4 Comparison to the Previous Computational Dimer Binding Estimates

Khare et al. (2006) have calculated the change in dimer binding stability from apo WT using *in silico* mutagenesis with MD in an implicit-solvation model. They find a ΔΔ*G* (A4V) ≈ − 11.0 kcal/mol and ΔΔ*G* (D101N) ≈ − 25.0 kcal/mol. These numbers are much larger than our numbers and display the reverse trend that D101N is significantly more destabilizing than A4V. We note that experimental measurements have shown that D101N is native-like in stability, as discussed above. In our calculations of the structural order in the *β*-barrel and loops 4 and 7, we found increased disorder of both the barrel and the loops in apo A4V monomer. This results in less stable interactions between the barrel and loops for this mutant, consistent with previous observations of the loss of specific contacts H71-L117 and H71-V118 based on short, 100 ns MD equilibrium simulations (Kumar et al., 2018b). Our observation of the increased *β*-barrel disorder in apo A4V monomer is consistent with previous observations of increased disorder specifically for strands *β*5-*β*6, based on short 60 ns equilibrium studies of A4V monomer using the *in lucem* Molecular Mechanics simulation software with an in-house force field (Tom et al., 2009).

We have previously calculated metal and dimer affinities for several SOD1 mutants (Das and Plotkin, 2013c). These previous calculations generated initial conditions by pulling monomers apart subject to distance and axis restraints via umbrella sampling and then implemented the weighted histogram analysis method (WHAM) (Shirts et al., 2007) to obtain a potential of mean force, similar to what has been implemented for smaller systems such as A*β* peptide (Lemkul and Bevan, 2010). However, this procedure is susceptible to convergence problems for our system and thus inaccuracies largely because of two problems related to conformational restrictions present when the protein is bound *versus* when it is free: 1) the orientational tumbling of each monomer relative to the other requires simulation timescales of ∼μs to be fully equilibrated (Wong and David, 2008) and thus does not generally reach equilibrium during the time of a typical MD simulation and 2) The available conformations and conformational entropy of each monomer are substantially increased when stabilizing dimer interface interactions are lost and the dimer is monomerized. This conformational relaxation may correspond to a very large free energy change and requires enhanced sampling techniques to properly evaluate. Each of these contributions significantly opposes binding, and their proper treatment is essential to accurately calculate the binding free energy.

Based on the present analysis, we infer that previous calculations of SOD1 dimer binding free energy have not been sufficiently systematic to calculate accurate numbers. Das and Plotkin (2013c) calculated approximately −15 kcal/mol for the dimer binding free energy of E,E (SS) SOD1, only slightly less binding free energy (−14.7 kcal/mol) for the E,E (SH) dimer, − 11.3 kcal/mol for apo A4V, and substantially more for WT Cu,Zn(SS) SOD1 (−25 kcal/mol). Although the values correlate reasonably well (*r* = 0.82) with the values obtained in this work, there is not enough data for statistical significance, and the magnitudes of the values are significantly different. We also reach qualitatively different conclusions in the present analysis, as, here, we find E,E (SS) A4V and E,E (SH) WT to be unstable.

### 4.5 The Choice of Coordinate System, Particularly r*, Adds an Arbitrary Element to the Method

We note that, in the coordinate system used here and in previous studies (Woo and Roux, 2005; Gumbart et al., 2013a; Sun et al., 2014; Prakash et al., 2017; Sayyed-Ahmad et al., 2016; Fu et al., 2017; Ulucan et al., 2014; Deng et al., 2018; Zeller and Zacharias, 2014; Lai and Kaznessis, 2017; Gumbart et al., 2013b; Zhang et al., 2018; Lahey and Rowley, 2020; Heinzelmann et al., 2017; Fu et al., 2018), the phase space explored under the restraints of the potential $u_{a}\left(\theta ,\phi \right)=\frac{1}{2}k_{a}\left({\left(\theta -\theta_{o}\right)}^{2}+{\left(\phi -\phi_{o}\right)}^{2}\right)$ increases as $∼{\left(r\left(\bar{P_{1}P_{1}^{\prime }}\right)\right)}^{2}$ . For this reason, there is some arbitrariness as to what distance this potential should be calculated. In practice, this variance is small. Variations in distance of 1 nm from the distance we use in this article (3.88 nm with corresponding average $\Delta G_{\text{dist}+\text{a}}^{\text{restr}}$ value of 21.20 kcal/mol) give an asymmetric “error” in free energy of $21.20_{-0.35}^{+0.27}$ kcal/mol.

## 5 Conclusion

The method we have used here, developed by Roux and colleagues, is the most systematic method available to find dimer binding free energies, and it is in principle exact although computationally expensive to implement. The calculated binding free energies for the SOD1 variants studied here are as follows: Δ*G*_WT Cu,Zn(SS)_ = − 5.0 ± 2.5 kcal/mol, Δ*G*_WT E,E(SS)_ = − 3.5 ± 2.9 kcal/mol, Δ*G*_WT E,E(SH)_ = + 1.0 ± 0.9 kcal/mol, Δ*G*_A4V E,E(SS)_ = + 2.3 ± 1.7 kcal/mol, and Δ*G*_D101 NE,E(SS)_ = − 6.7 ± 1.4 kcal/mol. These numbers differ quantitatively from the experimental values obtained for these variants: $\Delta G_{\text{WT E,E}\left(\text{SS}\right)}^{\exp}$ between −12 kcal/mol^52^ and −10 kcal/mol^18^, $\Delta G_{\text{WT E,E}\left(\text{SH}\right)}^{\exp}$ between −1 kcal/mol and −2 kcal/mol based on dissociation constants of transient populations (Sekhar et al., 2015), and $\Delta G_{\text{A}4\text{V E,E}\left(\text{SS}\right)}^{\exp}$ between −7.9 kcal/mol^18^ and −4 kcal/mol^16^, $\Delta G_{\text{D}101\text{N E,E}\left(\text{SS}\right)}^{\exp}=-12$ kcal/mol^8^. Our results do have the same trends seen in experiments in that WT E,E (SH) is marginally stable in the dimer, A4V E,E (SS) has reduced dimer stability from WT, metalation significantly increases the dimer stability, and the ALS-associated mutant D101N E,E (SS) has significant stability comparable with WT.

The computational method used here permits dissection of the contributions to the binding free energy. For all variants, there is a large penalty for dimer formation arising from the conformational entropy of disordered loops 4 and 7 in SOD1. The loop free energy penalty opposing dimerization is still significant even for the holoprotein, in spite of the increased loop ordering induced by bound metal cations. The apo A4V mutant has an unstable dimer due to weakened monomer-monomer interactions and increased flexibility of *β*-barrel in the free monomer for this mutant. Weakened inter-monomer interactions are manifested in the calculation as a smaller barrier height in the separation potential of mean force. On the contrary, D101N has a stable dimer partially due to an unusually rigid *β*-barrel in the free monomer.

In decomposing the contributions to the binding free energy, we have found several additional conclusions: disulfide reduction mainly affects the loop entropy contribution to dimer stability, the D101N mutation mainly affects the loop and barrel backbone entropy contribution to dimer stability, and variants that affect the loop regions [D101N E,E (SS) and WT E,E (SH)] disrupt the cooperative folding of loops in the native dimer.

It is an interesting future direction to check the consistency of this method using free energy alchemy for select mutants. The method we have used here also allows for non-perturbative effects, such as disulfide bond reduction or mispairing, large-scale evolutionary sequence differences, or nonsense mutants resulting in non-native sequence and/or an early stop codon. The present method also allows for *ab initio* absolute values rather than changes due to mutation. With sufficient computing resources, the accuracy of a given force field may be tested and validated using this method. These are the first applications of this systematic method to SOD1 dimer binding and are presently the most accurate computational predictions of SOD1 dimer binding free energy to date.

## Data Availability

The datasets presented in this study can be found in online repositories. The names of the repository/repositories and accession number(s) can be found at: https://phas.ubc.ca/∼steve/Dimer/.

## References

[B1] Abdolvahabi A. Shi Y. Rasouli S. Croom C. M. Aliyan A. Martí A. A. (2017). Kaplan-meier Meets Chemical Kinetics: Intrinsic Rate of SOD1 Amyloidogenesis Decreased by Subset of ALS Mutations and Cannot Fully Explain Age of Disease Onset. ACS Chem. Neurosci. 8 (6), 1378–1389. 10.1021/acschemneuro.7b00029 28290665

[B2] Abraham M. J. Murtola T. Schulz R. Páll S. Smith J. C. Hess B. (2015). GROMACS: High Performance Molecular Simulations through Multi-Level Parallelism from Laptops to Supercomputers. SoftwareX 1, 19–25. 10.1016/j.softx.2015.06.001

[B3] Ahalawat N. Mondal J. (2018). Assessment and Optimization of Collective Variables for Protein Conformational Landscape: GB1 *β*-hairpin as a Case Study. J. Chem. Phys. 149 (9), 094101. 10.1063/1.5041073 30195312

[B4] Ahl M. Lindberg M. J. Tibell L. A. E. (2004). Coexpression of Yeast Copper Chaperone (yCCS) and CuZn-Superoxide Dismutases in *Escherichia coli* Yields Protein with High Copper Contents. Protein Expr. Purif. 37 (2), 311–319. 10.1016/j.pep.2004.06.006 15358352

[B5] Ahmad G. Strange R. W. Whitson L. J. Antonyuk V. S. Narayana N. Taylor A. B. (2009). Structural and Biophysical Properties of Metal-free Pathogenic Sod1 Mutants A4V and G93A. Arch. Biochem. Biophys. 492 (1-2), 40–47. 10.1016/j.abb.2009.09.020 19800308 PMC2787720

[B6] Ahmed A. Rippmann F. Barnickel G. Gohlke H. (2011). A normal Mode-Based Geometric Simulation Approach for Exploring Biologically Relevant Conformational Transitions in Proteins. J. Chem. Inf. Model. 51 (7), 1604–1622. 10.1021/ci100461k 21639141

[B7] Andersen P. M. (2000). Genetic Factors in the Early Diagnosis of ALS. Amyotroph. Lateral Scler. Other Motor Neuron Disord. 1 (Suppl. 1), S31–S42. 10.1080/14660820052415899 11464924

[B8] Arnesano F. Banci L. Bertini I. Martinelli M. Furukawa Y. Thomas O’Halloran V. (2004). The Unusually Stable Quaternary Structure of Human Cu, Zn-Superoxide Dismutase 1 Is Controlled by Both Metal Occupancy and Disulfide Status. J. Biol. Chem. 279 (46), 47998–48003. 10.1074/jbc.m406021200 15326189

[B9] Banci L. Bertini I. Cantini F. D’Amelio N. Gaggelli E. (2006). Human SOD1 before Harboring the Catalytic Metal: Solution Structure of Copper-Depleted, Disulfide-Reduced Form. J. Biol. Chem. 281 (4), 2333–2337. 10.1074/jbc.m506497200 16291742

[B10] Banci L. Bertini I. Cramaro F. Del Conte R. Viezzoli M. S. (2003). Solution Structure of Apo Cu, Zn Superoxide Dismutase: Role of Metal Ions in Protein Folding. Biochemistry 42 (32), 9543–9553. 10.1021/bi034324m 12911296

[B11] Banci L. Bertini I. Cramaro F. Del Conte R. Viezzoli M. S. (2002). The Solution Structure of Reduced Dimeric Copper Zinc Superoxide Dismutase: the Structural Effects of Dimerization. Eur. J. Biochem. 269 (7), 1905–1915. 10.1046/j.1432-1033.2002.02840.x 11952792

[B12] Batey S. Randles L. G. Steward A. Clarke J. (2005). Cooperative Folding in a Multi-Domain Protein. J. Mol. Biol. 349 (5), 1045–1059. 10.1016/j.jmb.2005.04.028 15913648

[B13] Best R. B. Mittal J. (2010). Balance between *α* and *β* Structures in Ab Initio Protein Folding. The J. Phys. Chem. B 114 (26), 8790–8798. 10.1021/jp102575b 20536262

[B14] Bonomi M. (2019). Promoting Transparency and Reproducibility in Enhanced Molecular Simulations. Nat. Methods 16 (8), 670–673. 10.1038/s41592-019-0506-8 31363226

[B15] Boresch S. Tettinger F. Leitgeb M. Karplus M. (2003). Absolute Binding Free Energies: a Quantitative Approach for Their Calculation. J. Phys. Chem. B 107 (35), 9535–9551. 10.1021/jp0217839

[B16] Broom H. R. Rumfeldt J. A. O. Vassall K. A. Meiering E. M. (2015). Destabilization of the Dimer Interface Is a Common Consequence of Diverse ALS-Associated Mutations in Metal Free Sod1. Protein Sci. 24 (12), 2081–2089. 10.1002/pro.2803 26362407 PMC4815230

[B17] Broom H. R. Rumfeldt J. A. O. Vassall K. A. Meiering E. M. (2015). Destabilization of the Dimer Interface Is a Common Consequence of Diverse ALS-Associated Mutations in Metal Free SOD1. Protein Sci. 24 (12), 2081–2089. 10.1002/pro.2803 26362407 PMC4815230

[B18] Bruns C. K. Kopito R. R. (2007). Impaired post-translational Folding of Familial ALS-Linked Cu, Zn Superoxide Dismutase Mutants. EMBO J. 26 (3), 855–866. 10.1038/sj.emboj.7601528 17255946 PMC1794386

[B19] Byström R. Andersen P. M. Gröbner G. Oliveberg M. (2010). SOD1 Mutations Targeting Surface Hydrogen Bonds Promote Amyotrophic Lateral Sclerosis without Reducing Apo-State Stability. J. Biol. Chem. 285 (25), 19544–19552. 10.1074/jbc.m109.086074 20189984 PMC2885233

[B20] Cao X. Antonyuk V. S. Seetharaman S. V. Whitson L. J. Taylor A. B. Holloway S. P. (2008). Structures of the G85R Variant of SOD1 in Familial Amyotrophic Lateral Sclerosis. J. Biol. Chem. 283 (23), 16169–16177. 10.1074/jbc.m801522200 18378676 PMC2414278

[B21] Chantadul V. GarethWright S. A. Amporndanai K. Shahid M. V Antonyuk S. Gina W. (2020). Ebselen as Template for Stabilization of A4V Mutant Dimer for Motor Neuron Disease Therapy. Commun. Biol. 3 (1), 1–10. 10.1038/s42003-020-0826-3 32139772 PMC7058017

[B22] Christ C. D. Fox T. (2014). Accuracy Assessment and Automation of Free Energy Calculations for Drug Design. J. Chem. Inf. Model. 54 (1), 108–120. 10.1021/ci4004199 24256082

[B23] Conn A. R. Gould N. I. M. Toint P. L. (2000). Trust Region Methods. Philadelphia: SIAM.

[B24] Conway P. Tyka M. D. Frank D. M. Konerding D. E. Baker D. (2014). Relaxation of Backbone Bond Geometry Improves Protein Energy Landscape Modeling. Protein Sci. 23 (1), 47–55. 10.1002/pro.2389 24265211 PMC3892298

[B25] Das A. Plotkin S. S. (2013). Mechanical Probes of SOD1 Predict Systematic Trends in Metal and Dimer Affinity of ALS-Associated Mutants. J. Mol. Biol. 425 (5), 850–874. 10.1016/j.jmb.2012.12.022 23291526

[B26] Das A. Plotkin S. S. (2013). Mechanical Probes of SOD1 Predict Systematic Trends in Metal and Dimer Affinity of ALS-Associated Mutants. J. Mol. Biol. 425 (5), 850–874. 10.1016/j.jmb.2012.12.022 23291526

[B27] Das A. Plotkin S. S. (2013). SOD1 Exhibits Allosteric Frustration to Facilitate Metal Binding Affinity. Proc. Natl. Acad. Sci. 110 (10), 3871–3876. 10.1073/pnas.1216597110 23431152 PMC3593857

[B28] Deng N. Cui Di. Zhang B. W. Xia J. Cruz J. Levy R. (2018). Comparing Alchemical and Physical Pathway Methods for Computing the Absolute Binding Free Energy of Charged Ligands. Phys. Chem. Chem. Phys. 20 (25), 17081–17092. 10.1039/c8cp01524d 29896599 PMC6061996

[B29] Doucette P. A. Whitson L. J. Cao X. Schirf V. Demeler B. Valentine J. S. (2004). Dissociation of Human Copper-Zinc Superoxide Dismutase Dimers Using Chaotrope and Reductant. J. Biol. Chem. 279 (52), 54558–54566. 10.1074/jbc.m409744200 15485869

[B30] Elam J. S. Malek K. Rodriguez J. A. Doucette P. A. Taylor A. B. Hayward L. J. (2003b). An Alternative Mechanism of Bicarbonate-Mediated Peroxidation by Copper-Zinc Superoxide Dismutase: Rates Enhanced via Proposed Enzyme-Associated Peroxycarbonate Intermediate. J. Biol. Chem. 278 (23), 21032–21039. 10.1074/jbc.m300484200 12649272

[B31] Elam J. S. Taylor A. B. Richard S. Antonyuk S. Doucette P. A. Rodriguez J. A. (2003a). Amyloid-like Filaments and Water-Filled Nanotubes Formed by Sod1 Mutant Proteins Linked to Familial ALS. Nat. Struct. Mol. Biol. 10 (6), 461–467. 10.1038/nsb935 12754496

[B32] Fajardo T. N. Heyden M. (2021). Dissecting the Conformational Free Energy of a Small Peptide in Solution. J. Phys. Chem. B 125 (18), 4634–4644. 10.1021/acs.jpcb.1c00699 33942611

[B33] Fajer M. Swift R. V. Mc Cammon J. A. (2009). Using Multistate Free Energy Techniques to Improve the Efficiency of Replica Exchange Accelerated Molecular Dynamics. J. Comput. Chem. 30 (11), 1719–1725. 10.1002/jcc.21285 19421994 PMC2700186

[B34] Farrell D. W. Speranskiy K. Thorpe M. F. (2010). Generating Stereochemically Acceptable Protein Pathways. Proteins: Struct. Funct. Bioinformatics 78 (14), 2908–2921. 10.1002/prot.22810 20715289

[B35] Frisch M. J. Trucks G. W. Schlegel H. B. Scuseria G. E. Robb M. A. Cheeseman J. R. (2009). Gaussian 09 Citation. Wallingford CT: gaussian. Inc., 201. revision d. 01.

[B36] Fu H. Cai W. Hénin J. Roux B. Chipot C. (2017). New Coarse Variables for the Accurate Determination of Standard Binding Free Energies. J. Chem. Theor. Comput. 13 (11), 5173–5178. 10.1021/acs.jctc.7b00791 28965398

[B37] Fu H. Gumbart J. C. Chen H. Shao X. Cai W. Chipot C. (2018). BFEE: A User-Friendly Graphical Interface Facilitating Absolute Binding Free-Energy Calculations. J. Chem. Inf. Model. 58 (3), 556–560. 10.1021/acs.jcim.7b00695 29405709 PMC5869121

[B38] Gathiaka S. Liu S. Chiu M. Yang H. Stuckey J. A. Kang Y. N. (2016). D3R Grand challenge 2015: Evaluation of Protein-Ligand Pose and Affinity Predictions. J. computer-aided Mol. Des. 30 (9), 651–668. 10.1007/s10822-016-9946-8 PMC5562487 27696240

[B39] Gee J. Scott Shell M. (2011). Two-dimensional Replica Exchange Approach for Peptide-Peptide Interactions. J. Chem. Phys. 134 (6), 02B619. 10.1063/1.3551576 21322666

[B40] Gruszka T. D. Mendonça C. A. T. F. Paci E. Whelan F. Hawkhead J. Potts J. R. (2016). Disorder Drives Cooperative Folding in a Multidomain Protein. Proc. Natl. Acad. Sci. 113 (42), 11841–11846. 10.1073/pnas.1608762113 27698144 PMC5081646

[B41] Gregorio Nivón L. Moretti R. Baker D. (2013). A Pareto-Optimal Refinement Method for Protein Design Scaffolds. PloS one 8 (4), e59004. 10.1371/journal.pone.0059004 23565140 PMC3614904

[B42] Gumbart J. C. Roux B. Chipot C. (2013). Standard Binding Free Energies from Computer Simulations: What Is the Best Strategy? J. Chem. Theor. Comput. 9 (1), 794–802. 10.1021/ct3008099 PMC3685508 23794960

[B43] Gumbart J. C. Roux B. Chipot C. (2013). Efficient Determination of Protein-Protein Standard Binding Free Energies from First Principles. J. Chem. Theor. Comput. 9 (8), 3789–3798. 10.1021/ct400273t PMC3809040 24179453

[B44] Habibi M. Plotkin S. S. Rottler J. (2018). Soft Vibrational Modes Predict Breaking Events during Force-Induced Protein Unfolding. Biophysical J. 114 (3), 562–569. 10.1016/j.bpj.2017.11.3781 PMC5985024 29414701

[B45] Habibi M. Rottler J. Plotkin S. S. (2016). As Simple as Possible, but Not Simpler: Exploring the Fidelity of Coarse-Grained Protein Models for Simulated Force Spectroscopy. PLoS Comput. Biol. 12 (11), e1005211. 10.1371/journal.pcbi.1005211 27898663 PMC5127490

[B46] Habibi M. Rottler J. Plotkin S. S. (2017). The Unfolding Mechanism of Monomeric Mutant SOD1 by Simulated Force Spectroscopy. BBA-Proteins and Proteomics 1865 (11), 1631–1642. 10.1016/j.bbapap.2017.06.009 28629863

[B47] Hansen N. WilfredGunsteren F. V. (2014). Practical Aspects of Free-Energy Calculations: a Review. J. Chem. Theor. Comput. 10 (7), 2632–2647. 10.1021/ct500161f 26586503

[B48] Hazel A. J. Walters E. T. Rowley C. N. Gumbart J. C. (2018). Folding Free Energy Landscapes of *β*-sheets with Non-polarizable and Polarizable CHARMM Force fields. J. Chem. Phys. 149 (7), 072317. 10.1063/1.5025951 30134731

[B49] Heinzelmann G. NielHenriksen M. Gilson M. K. (2017). Attach-pull-release Calculations of Ligand Binding and Conformational Changes on the First BRD4 Bromodomain. J. Chem. Theor. Comput. 13 (7), 3260–3275. 10.1021/acs.jctc.7b00275 PMC5541932 28564537

[B50] Hermans Jan. Shankar S. (1986). The Free Energy of Xenon Binding to Myoglobin from Molecular Dynamics Simulation. Isr. J. Chem. 27 (2), 225–227. 10.1002/ijch.198600032

[B51] Hörnberg A. Logan D. T. Marklund S. L. Oliveberg M. (2007). The Coupling between Disulphide Status, Metallation and Dimer Interface Strength in Cu/Zn Superoxide Dismutase. J. Mol. Biol. 365 (2), 333–342. 10.1016/j.jmb.2006.09.048 17070542

[B52] Hough M. A. Grossmann J. G. V Antonyuk S. Strange R. W. PeterDoucette A. Rodriguez J. A. (2004). Dimer Destabilization in Superoxide Dismutase May Result in Disease-Causing Properties: Structures of Motor Neuron Disease Mutants. Proc. Natl. Acad. Sci. 101 (16), 5976–5981. 10.1073/pnas.0305143101 15056757 PMC395908

[B87] Hsueh S.C. C. Plotkin S. S. (2022). Accelerated Ensemble Generation for Cyclic Peptide Using Reservoir-REMD. [Preprint]. 10.1021/acs.jpcb.2c05470 36410027

[B53] Huai J. Zhang Z. (2019). Structural Properties and Interaction Partners of Familial ALS-Associated SOD1 Mutants. Front. Neurol. 10, 527. 10.3389/fneur.2019.00527 31164862 PMC6536575

[B54] Huang J. Rauscher S. Nawrocki G. Ran T. Feig M. Bert L. (2017). Charmm36m: an Improved Force Field for Folded and Intrinsically Disordered Proteins. Nat. Methods 14 (1), 71–73. 10.1038/nmeth.4067 27819658 PMC5199616

[B55] Lahey J.S. Rowley C. N. (2020). Simulating Protein-Ligand Binding with Neural Network Potentials. Chem. Sci. 11 (9), 2362–2368. 10.1039/c9sc06017k 34084397 PMC8157423

[B56] Jorgensen W. L. Chandrasekhar J. Madura J. D. Impey R. W. Klein M. L. (1983). Comparison of Simple Potential Functions for Simulating Liquid Water. J. Chem. Phys. 79 (2), 926–935. 10.1063/1.445869

[B57] Joshi D. C. Lin J. -H. (2019). Delineating Protein-Protein Curvilinear Dissociation Pathways and Energetics with Naïve Multiple-walker Umbrella Sampling Simulations. J. Comput. Chem. 40 (17), 1652–1663. 10.1002/jcc.25821 30950525

[B58] Kayatekin C. Zitzewitz J. A. Matthews C. R. (2008). Zinc Binding Modulates the Entire Folding Free Energy Surface of Human Cu, Zn Superoxide Dismutase. J. Mol. Biol. 384 (2), 540–555. 10.1016/j.jmb.2008.09.045 18840448 PMC2756654

[B59] Kevin S. Sohn S. H. Durazo A. Sheng Y. Shaw B. F. Cao X. (2015). Insights into the Role of the Unusual Disulfide Bond in Copper-Zinc Superoxide Dismutase. J. Biol. Chem. 290 (4), 2405–2418. 10.1074/jbc.M114.588798 25433341 PMC4303690

[B60] Khare S. D. Caplow M. Dokholyan N. V. (2006). FALS Mutations in Cu, Zn Superoxide Dismutase Destabilize the Dimer and Increase Dimer Dissociation Propensity: a Large-Scale Thermodynamic Analysis. Amyloid 13 (4), 226–235. 10.1080/13506120600960486 17107883

[B61] Khatib F. Cooper S. Tyka M. D. Xu K. Makedon I. Popović Z. (2011). Algorithm Discovery by Protein Folding Game Players. Proc. Natl. Acad. Sci. 108 (47), 18949–18953. 10.1073/pnas.1115898108 22065763 PMC3223433

[B62] Kumar V. Prakash A. Lynn A. M. (2018b). Alterations in Local Stability and Dynamics of A4V SOD1 in the Presence of Trifluoroethanol. Biopolymers 109 (3), e23102. 10.1002/bip.23102 29369331

[B63] Kumar V. Prakash A. Pandey P. Lynn A. M. Hassan M. I. (2018a). TFE-induced Local Unfolding and Fibrillation of SOD1: Bridging the experiment and Simulation Studies. Biochem. J. 475 (10), 1701–1719. 10.1042/bcj20180085 29686043

[B64] Lai P.-K. Kaznessis Y. N. (2017). Free Energy Calculations of Microcin J25 Variants Binding to the Fhua Receptor. J. Chem. Theor. Comput. 13 (7), 3413–3423. 10.1021/acs.jctc.7b00417 PMC9481273 28622469

[B65] Leaver-Fay A. Tyka M. Lewis S. M. Lange O. F. Thompson J. Ron J. (2011). ROSETTA3: an Object-Oriented Software Suite for the Simulation and Design of Macromolecules. Methods Enzymol. 487, 545–574. 10.1016/b978-0-12-381270-4.00019-6 21187238 PMC4083816

[B66] Lee K.-H. Kuczera K. (2021). Free Energy Simulations to Understand the Effect of Met → Ala Mutations at Positions 205, 206 and 213 on Stability of Human Prion Protein. Biophysical Chem. 275, 106620. 10.1016/j.bpc.2021.106620 34058726

[B67] Lemkul J. A. Bevan D. R. (2010). Assessing the Stability of Alzheimer’s Amyloid Protofibrils Using Molecular Dynamics. J. Phys. Chem. B 114, 1652–1660. 10.1021/jp9110794 20055378

[B68] Lindberg M. J. Bystrom R. Boknas N. Andersen P. M. Oliveberg M. (2005). Systematically Perturbed Folding Patterns of Amyotrophic Lateral Sclerosis (Als)-associated Sod1 Mutants. Proc. Natl. Acad. Sci. 102, 9754–9759. 10.1073/pnas.0501957102 15987780 PMC1174986

[B69] Lindberg M. J. Tibell L. Oliveberg M. (2002). Common Denominator of Cu/zn Superoxide Dismutase Mutants Associated with Amyotrophic Lateral Sclerosis: Decreased Stability of the Apo State. Proc. Natl. Acad. Sci. 99 (26), 16607–16612. 10.1073/pnas.262527099 12482932 PMC139191

[B70] Lindorff-Larsen K. Paul M. Piana S. Eastwood M. P. Dror R. O. Shaw D. E. (2012). Systematic Validation of Protein Force fields against Experimental Data. PloS one 7 (2), e32131. 10.1371/journal.pone.0032131 22384157 PMC3285199

[B71] Maple J. R. Dinur U. Hagler A. T. (1988). Derivation of Force fields for Molecular Mechanics and Dynamics from Ab Initio Energy Surfaces. Proc. Natl. Acad. Sci. 85 (15), 5350–5354. 10.1073/pnas.85.15.5350 16593959 PMC281753

[B72] McAlary L. Yerbury J. J. Aquilina J. A. (2013). Glutathionylation Potentiates Benign Superoxide Dismutase 1 Variants to the Toxic Forms Associated with Amyotrophic Lateral Sclerosis. Sci. Rep. 3, 3275. 10.1038/srep03275 24253732 PMC3834562

[B73] Lindberg M. J. Normark J. Holmgren A. Oliveberg M. (2004). Folding of Human Superoxide Dismutase: Disulfide Reduction Prevents Dimerization and Produces Marginally Stable Monomers. Proc. Natl. Acad. Sci. 101 (45), 15893–15898. 10.1073/pnas.0403979101 15522970 PMC528748

[B74] Okur A. Roe D. R. Cui G. Hornak V. Simmerling C. (2007). Improving Convergence of Replica-Exchange Simulations through Coupling to a High-Temperature Structure Reservoir. J. Chem. Theor. Comput. 3 (2), 557–568. 10.1021/ct600263e 26637035

[B75] Peng X. Cashman N. R. Plotkin S. S. (2018). Prediction of Misfolding-specific Epitopes in Sod1 Using Collective Coordinates. J. Phys. Chem. B 122 (49), 11662–11676. 10.1021/acs.jpcb.8b07680 30351123

[B76] Piana S. Klepeis J. L. Shaw D. E. (2014). Assessing the Accuracy of Physical Models Used in Protein-Folding Simulations: Quantitative Evidence from Long Molecular Dynamics Simulations. Curr. Opin. Struct. Biol. 24, 98–105. 10.1016/j.sbi.2013.12.006 24463371

[B77] Piana S. Lindorff-Larsen K. Shaw D. E. (2011). How Robust Are Protein Folding Simulations with Respect to Force Field Parameterization? Biophysical J. 100 (9), L47–L49. 10.1016/j.bpj.2011.03.051 PMC3149239 21539772

[B78] Prakash P. Sayyed-Ahmad A. Cho K.-J. Dolino D. M. Chen W. Li H. (2017). Computational and Biochemical Characterization of Two Partially Overlapping Interfaces and Multiple Weak-Affinity K-Ras Dimers. Scientific Rep. 7 (1), 1–11. 10.1038/srep40109 PMC5220301 28067274

[B79] Prudencio M. Hart P. J. Borchelt D. R. Andersen P. M. (2009). Variation in Aggregation Propensities Among ALS-Associated Variants of SOD1: Correlation to Human Disease. Hum. Mol. Genet. 18 (17), 3217–3226. 10.1093/hmg/ddp260 19483195 PMC2722984

[B80] Roberts B. R. Tainer J. A. Getzoff E. D. Malencik D. A. Anderson S. R. Bomben V. C. (2007). Structural Characterization of Zinc-Deficient Human Superoxide Dismutase and Implications for ALS. J. Mol. Biol. 373 (4), 877–890. 10.1016/j.jmb.2007.07.043 17888947 PMC2175016

[B81] Rodriguez J. A. Shaw B. F. Durazo A. Sohn S. H. Doucette P. A. Nersissian A. M. (2005). Destabilization of Apoprotein Is Insufficient to Explain Cu, Zn-Superoxide Dismutase-Linked ALS Pathogenesis. Proc. Natl. Acad. Sci. 102 (30), 10516–10521. 10.1073/pnas.0502515102 16020530 PMC1175580

[B82] Rogers J. M. (2020). Peptide Folding and Binding Probed by Systematic Non-canonical Mutagenesis. Front. Mol. Biosciences 7, 100. 10.3389/fmolb.2020.00100 PMC7326784 32671094

[B83] Sandelin E. Nordlund A. Andersen P. M. Marklund S. L. Oliveberg M. (2007). Amyotrophic Lateral Sclerosis-Associated Copper/zinc Superoxide Dismutase Mutations Preferentially Reduce the Repulsive Charge of the Proteins. J. Biol. Chem. 282 (29), 21230–21236. 10.1074/jbc.m700765200 17513298

[B84] Sayyed-Ahmad A. Cho K. -J. Hancock J. F. Gorfe A. A. (2016). Computational Equilibrium Thermodynamic and Kinetic Analysis of K-Ras Dimerization through an Effector Binding Surface Suggests Limited Functional Role. J. Phys. Chem. B 120 (33), 8547–8556. 10.1021/acs.jpcb.6b02403 27072779 PMC5048749

[B85] Sekhar A. Rumfeldt J. A. O. Broom H. R. Doyle C. M. Bouvignies G. Meiering E. M. (2015). Thermal Fluctuations of Immature Sod1 lead to Separate Folding and Misfolding Pathways. eLife 4, e07296. 10.7554/eLife.07296 26099300 PMC4475725

[B86] Semmler S. Gagné M. Garg P. SarahPickles R. Baudouin C. Hamon-Keromen E. (2020). Tnf Receptor-Associated Factor 6 Interacts with Als-Linked Misfolded Superoxide Dismutase 1 and Promotes Aggregation. J. Biol. Chem. 295 (12), 3808–3825. 10.1074/jbc.ra119.011215 32029478 PMC7086032

[B88] Shi Y. Acerson M. J. Abdolvahabi A. Mowery R. A. Shaw B. F. (2016). Gibbs Energy of Superoxide Dismutase Heterodimerization Accounts for Variable Survival in Amyotrophic Lateral Sclerosis. J. Am. Chem. Soc. 138 (16), 5351–5362. 10.1021/jacs.6b01742 27054659

[B89] Shirts M. R. Chodera J. D. (2008). Statistically Optimal Analysis of Samples from Multiple Equilibrium States. J. Chem. Phys. 129 (12), 124105. 10.1063/1.2978177 19045004 PMC2671659

[B90] Shirts M. R. Mobley D. L. Chodera J. D. (2007). Chapter 4 Alchemical Free Energy Calculations: Ready for Prime Time? Annu. Rep. Comput. Chem. 3, 41–59. 10.1016/s1574-1400(07)03004-6

[B91] Siebenmorgen T. Zacharias M. (2019). Evaluation of Predicted Protein-Protein Complexes by Binding Free Energy Simulations. J. Chem. Theor. Comput. 15 (3), 2071–2086. 10.1021/acs.jctc.8b01022 30698954

[B92] Stathopulos P. B. Rumfeldt J. A. O. Scholz G. A. Irani R. A. He Frey. Hallewell R. A. Lepock J. R. (2003). Cu/Zn Superoxide Dismutase Mutants Associated with Amyotrophic Lateral Sclerosis Show Enhanced Formation of Aggregates *In Vitro* . Proc. Natl. Acad. Sci. 100 (12), 7021–7026. 10.1073/pnas.1237797100 12773627 PMC165823

[B93] Strange R. W. Antonyuk S. Hough M. A. Doucette P. A. Rodriguez J. A. Hart P. J. (2003). The Structure of Holo and Metal-Deficient Wild-type Human Cu, Zn Superoxide Dismutase and its Relevance to Familial Amyotrophic Lateral Sclerosis. J. Mol. Biol. 328 (4), 877–891. 10.1016/s0022-2836(03)00355-3 12729761

[B94] Sun H. Li Y. Tian S. Wang J. Hou T. (2014). P-loop Conformation Governed Crizotinib Resistance in G2032R-Mutated ROS1 Tyrosine Kinase: Clues from Free Energy Landscape. PLoS Comput. Biol. 10 (7), e1003729. 10.1371/journal.pcbi.1003729 25033171 PMC4102447

[B95] Svensson A. K. E. Bilsel O. Kayatekin C. Adefusika J. A. Zitzewitz J. A. Matthews C. R. (2010). Metal-free ALS Variants of Dimeric Human Cu, Zn-Superoxide Dismutase Have Enhanced Populations of Monomeric Species. PLoS One 5 (4), e10064. 10.1371/journal.pone.0010064 20404910 PMC2852398

[B96] Tan Z. Gallicchio E. Lapelosa M. Levy R. M. (2012). Theory of Binless Multi-State Free Energy Estimation with Applications to Protein-Ligand Binding. J. Chem. Phys. 136 (14), 04B608. 10.1063/1.3701175 PMC3339880 22502496

[B97] Tiwari A. Liba A. Sohn S. H. Seetharaman S. V. Bilsel O. Matthews C. R. (2009). Metal Deficiency Increases Aberrant Hydrophobicity of Mutant Superoxide Dismutases that Cause Amyotrophic Lateral Sclerosis. J. Biol. Chem. 284 (40), 27746–27758. 10.1074/jbc.m109.043729 19651777 PMC2785702

[B98] Tom S. Kennedy B. K. Daggett V. (2009). Structural Changes to Monomeric CuZn Superoxide Dismutase Caused by the Familial Amyotrophic Lateral Sclerosis-Associated Mutation A4V. Biophysical J. 97 (6), 1709–1718. 10.1016/j.bpj.2009.06.043 PMC2749781 19751676

[B99] Tyka M. D. Keedy D. A. André I. Frank D. M. Song Y. Richardson D. C. (2011). Alternate States of Proteins Revealed by Detailed Energy Landscape Mapping. J. Mol. Biol. 405 (2), 607–618. 10.1016/j.jmb.2010.11.008 21073878 PMC3046547

[B100] UBC ARC Sockeye (2022). UBC Advanced Research Computing. Available at: https://arc.ubc.ca/ubc-arc-sockeye (Accessed February 28, 2022).

[B101] Ulucan O. Jaitly T. Helms V. (2014). Energetics of Hydrophilic Protein-Protein Association and the Role of Water. J. Chem. Theor. Comput. 10 (8), 3512–3524. 10.1021/ct5001796 26588315

[B102] Valentine J. S. Doucette P. A. Potter S. Z. (2005). Copper-Zinc Superoxide Dismutase and Amyotrophic Lateral Sclerosis. Annu. Rev. Biochem. 74 (1), 563–593. 10.1146/annurev.biochem.72.121801.161647 15952898

[B103] Vassall K. A. PeterStathopulos B. Rumfeldt J. A. O. JamesLepock R. Meiering E. M. (2006). Equilibrium Thermodynamic Analysis of Amyotrophic Lateral Sclerosis-Associated Mutant Apo Cu, Zn Superoxide Dismutases. Biochemistry 45 (23), 7366–7379. 10.1021/bi0600953 16752926

[B104] Waldher B. Kuta J. Chen S. Henson N. Clark A. E. (2010). ForceFit: A Code to Fit Classical Force fields to Quantum Mechanical Potential Energy Surfaces. J. Comput. Chem. 31 (12), 2307–2316. 10.1002/jcc.21523 20340109

[B105] Walther Perthold J. Oostenbrink C. (2019). GroScore: Accurate Scoring of Protein-Protein Binding Poses Using Explicit-Solvent Free-Energy Calculations. J. Chem. Inf. Model. 59 (12), 5074–5085. 10.1021/acs.jcim.9b00687 31790223

[B106] Wang Q. Johnson J. L. Agar N. Y. R. Agar J. N. (2008). Protein Aggregation and Protein Instability Govern Familial Amyotrophic Lateral Sclerosis Patient Survival. Plos Biol. 6 (7), e170. 10.1371/journal.pbio.0060170 18666828 PMC2486295

[B107] Wells N. G. M. Tillinghast G. A. O’Neil A. L. Smith C. A. (2021). Free Energy Calculations of ALS-Causing SOD1 Mutants Reveal Common Perturbations to Stability and Dynamics along the Maturation Pathway. Protein Sci. 30, 1804–1817. 10.1002/pro.4132 34076319 PMC8376412

[B108] Wong V. David A. (2008). Case. Evaluating Rotational Diffusion from Protein MD Simulations. J. Phys. Chem. B 112 (19), 6013–6024. 10.1021/jp0761564 18052365

[B109] Woo H.-J. Roux B. (2005). Calculation of Absolute Protein-Ligand Binding Free Energy from Computer Simulations. Proc. Natl. Acad. Sci. 102 (19), 6825–6830. 10.1073/pnas.0409005102 15867154 PMC1100764

[B110] Wroe R. Butler A. W. L. Andersen P. M. Powell J. F. Al-Chalabi A. (2008). ALSOD: the Amyotrophic Lateral Sclerosis Online Database. Amyotroph. Lateral Scler. 9 (4), 249–250. 10.1080/17482960802146106 18608099

[B111] Zeller F. Zacharias M. (2014). Evaluation of Generalized Born Model Accuracy for Absolute Binding Free Energy Calculations. J. Phys. Chem. B 118 (27), 7467–7474. 10.1021/jp5015934 24941018

[B112] Zhang H. Gattuso H. Dumont E. Cai W. Monari A. Chipot C. (2018). Accurate Estimation of the Standard Binding Free Energy of Netropsin with DNA. Molecules 23 (2), 228. 10.3390/molecules23020228 PMC6017086 29370096

[B113] Zhang Z. Santos A. P. Zhou Q. Liang L. Wang Q. Wu T. (2016). Steered Molecular Dynamics Study of Inhibitor Binding in the Internal Binding Site in Dehaloperoxidase-Hemoglobin. Biophysical Chem. 211, 28–38. 10.1016/j.bpc.2016.01.003 26824426

