# Supplementary material for: First Principles Calculation of Protein–Protein Dimer Affinities of ALS-Associated SOD1 Mutants
Source: Front Mol Biosci. 2022 Mar 24;9:845013. doi: 10.3389/fmolb.2022.845013 (PMC8988244; doi:10.3389/fmolb.2022.845013)
Supplement: Supplementary file 1 [file DataSheet1.pdf]

Supplementary Material for:

First principles calculation of protein-protein dimer affinities of ALS-Associated SOD1 Mutants

Shawn C.C. Hsueh[1], Mark Nijland[1,2,3], Xubiao Peng[1,4], Benjamin Hilton[1,5], and Steven S. Plotkin*[1,6]

[1]Department of Physics and Astronomy, University of British Columbia, Vancouver, BC, Canada
[2]Laboratory of Organic Chemistry, Wageningen University and Research, Wageningen, Netherlands
[3]Laboratory of Physical Chemistry and Soft Matter, Wageningen University and Research, Wageningen, Netherlands
[4]Center for QuantumTechnology Research, School of Physics, Beijing Institute of Technology, Beijing, China
[5]Imperial College London, London, United Kingdom
[6]Science and Technology Program, University of British Columbia, Vancouver, BC, Canada

March 16, 2022

S1 Error analysis for each energy contribution

S1.1 S^* and ΔG_o^{free}

Since S^* and ΔG_o^{free} are calculated analytically, there are no error associate with the two terms.

S1.2 $\Delta G_{\text{dist+a}}^{\text{restr}}$

The integral of the PMF in Eq. (7) (main text) is dominated by the immediate vicinity of the distance in the bound state. Since the PMF error at r^* is typically much larger than the PMF error at the bound state (Fig. 3 (main text) right most column), the error of I^* could be safely approximated by the error propagation of $W(r^*)$ alone (Eq. (S1)).

$$\sigma_{I^*}^2 = \left(\frac{\partial I^*}{\partial W(r^*)}\right)^2 \left(\sigma_{W(r^*)}\right)^2 = \left(\beta I^* \sigma_{W(r^*)}\right)^2 \tag{S1}$$

where the error $\sigma_{W(r^*)}$ is taken from the larger value of **1)** statistical error of MBAR estimator calculated from the last 50% REMD-US trajectories, or **2)** the difference of $W(r^*)$ calculated from the last 75% and the last 50% REMD-US trajectories. The error of $\Delta G_{\text{dist+a}}^{\text{restr}}$ is then given by the simple expression:

$$\sigma^2_{\Delta G_{\text{dist+a}}^{\text{restr}}} = \left(\frac{\partial \Delta G_{\text{dist+a}}^{\text{restr}}}{\partial I^*}\right)^2 \sigma_{I^*}^2 = \frac{\sigma_{I^*}^2}{\beta^2 I^{*2}} = \sigma_{W(r^*)}^2 \tag{S2}$$

S1.3 Other contributions to Eq. (11) (main text)

The error of the other free energy contributions in Eq. (11) (main text) are approximated by the larger of two values, as obtained from either of the following two methods:

1) The standard deviation of free energy perturbation calculation from 10,000 re-samplings of the PMF. The re-sampled PMF at certain reaction coordinate value, ξ_i, is sampled from a Gaussian probability function

$$P_{\xi_i}(F) = exp\left(-\frac{(F - F_0(\xi_i))}{2\sigma^2_{F_0(\xi_i)}}\right) \tag{S3}$$

where $F_0(\xi_i)$ and $\sigma_{F_0(\xi_i)}$ are the MBAR estimated PMF value and error at ξ_i.

2) The difference of the free energy contribution (e.g. $\Delta G_{LA,c}^{\text{bound}}$) calculated by the last 75% and the last 50% REMD-US trajectories.

S2 Data sets

All reaction coordinate trajectories in biasing umbrella potentials (files named `Umbrella.txt`) and umbrella bias potential files (`plumed.dat`), are available in a compressed .tar.gz file at
`https://phas.ubc.ca/~steve/Dimer/`
These files are sufficient to construct the PMFs in the present work. The five folders named `EESS, EESH, EESS_A4V, EESS_D101N`, and `CuZnSS` contain the data for five respective SOD1 variants (in the same order as in Table 5 (main text)). For each variant, there are 16 subfolders:

- 6 subfolders are for conformational contributions in the dimer (`C_LA, C_LB, C_BA, C_BB, C_IA`, and `C_IB`),
- 5 subfolders are for orientational/angular contribution in the dimer (`O_THETA, O_PHI, O_PSI, A_theta`, and `A_phi`),
- 3 subfolders are for conformational contributions in the monomer (`C_L, C_B`, and `C_I`),
- 1 subfolder is for the separation PMF (`I`),

• and 1 subfolder is for the equilibration (`equil`).

In each folder that represents free energy contribution (folders other than `equil`), there is a folder `serial` and a folder `RE`, which store the data for initial configuration preparation and REMD-US. The reaction coordinate trajectory of each umbrella in REMD-US simulation is stored in a folder named starting with "Z" followed by the bias center (units are in either nm or rad) of the reaction coordinate (e.g. `Z0.52` in `C_L` corresponds to an umbrella with bias center at RMSD= 0.52 nm).

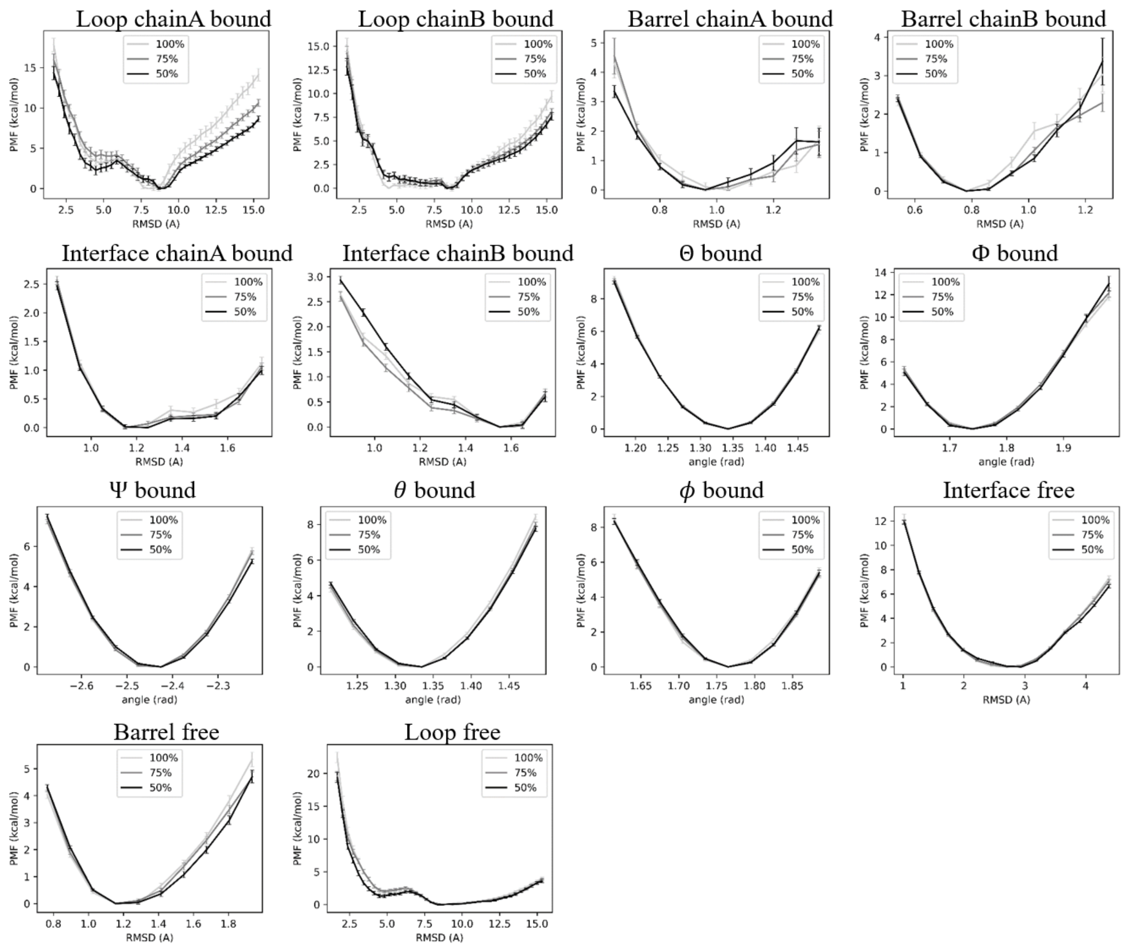

Figure S1: **The PMFs for different fractions of the MD trajectories for WT E,E(SS), to illustrate convergence.** PMFs are constructed with either 100%, the last 75%, or the last 50% of the REMD-US trajectories, to show convergence. The PMFs shown in each panel are ordered from the top-left to the lower-right in the same order that the restraints are sequentially applied or released, following the arrow in Fig. 2 (main text).

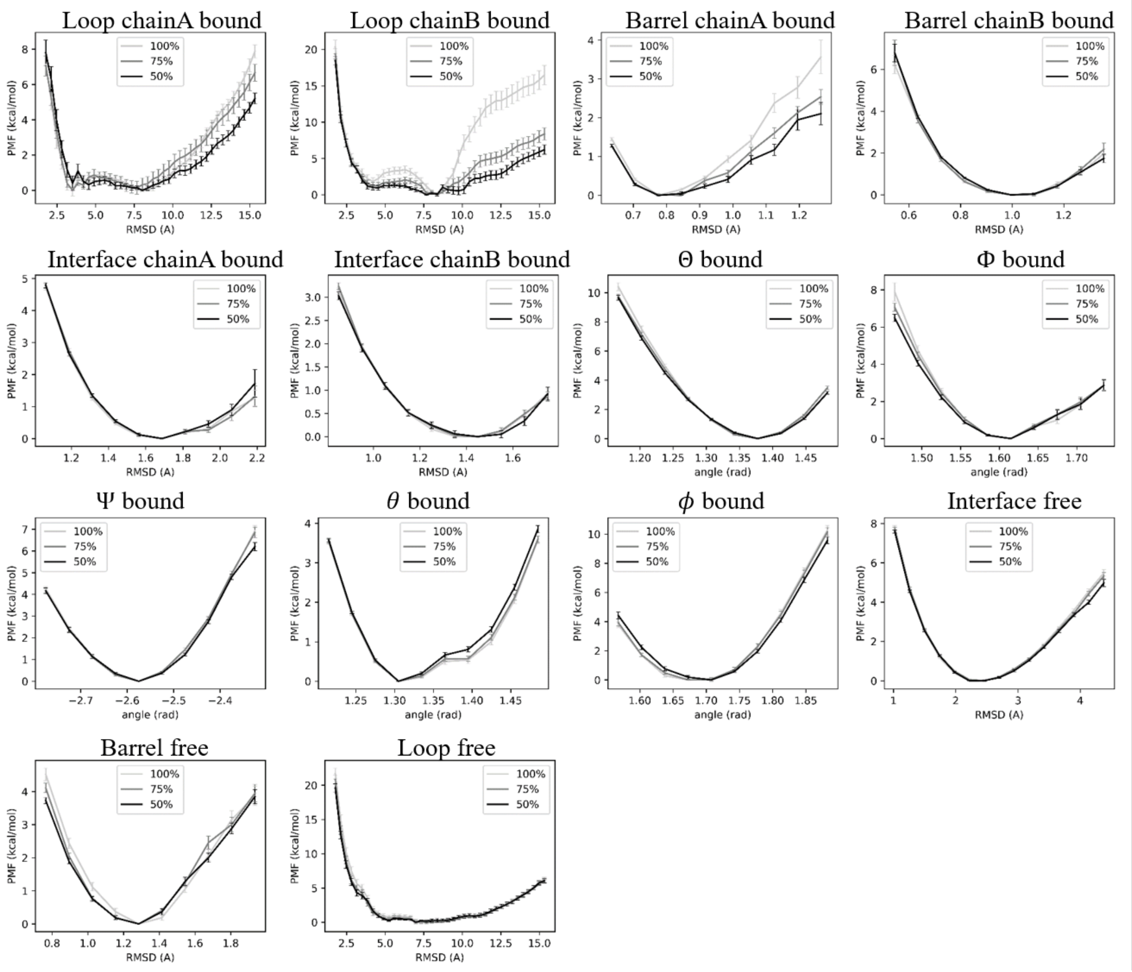

Figure S2: **The PMFs for different fractions of the MD trajectories for WT E,E(SH), to illustrate convergence.** PMFs constructed with the full, the last 75%, and the last 50% of the REMD-US trajectories are calculated to show the convergence. The PMFs from the top-left to the lower-right are with incremental/decremental restraints, and follows the arrow in Fig. 2 (main text).

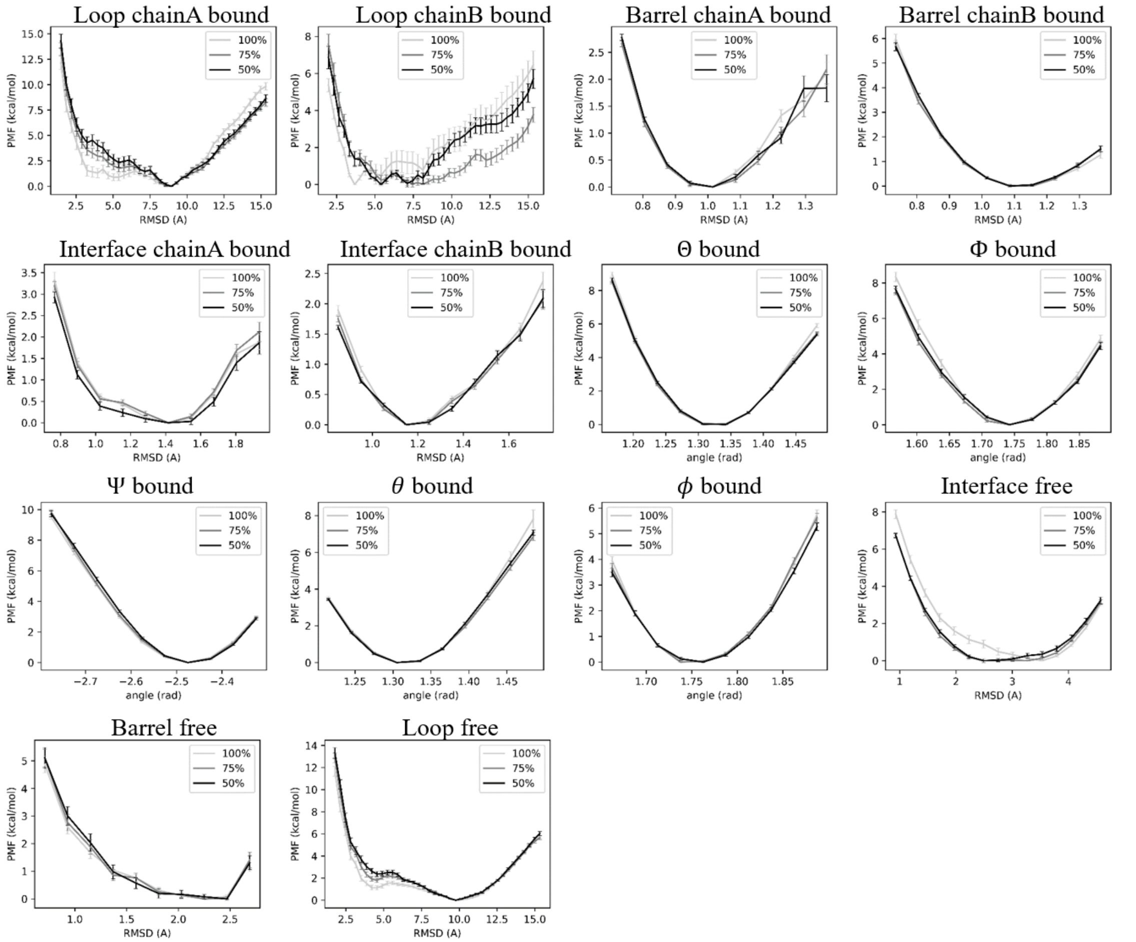

Figure S3: **The PMFs for different fractions of the MD trajectories for A4V E,E(SS), to illustrate convergence.** PMFs constructed with the full, the last 75%, and the last 50% of the REMD-US trajectories are calculated to show the convergence. The PMFs from the top-left to the lower-right are with incremental/decremental restraints, and follows the arrow in Fig. 2 (main text).

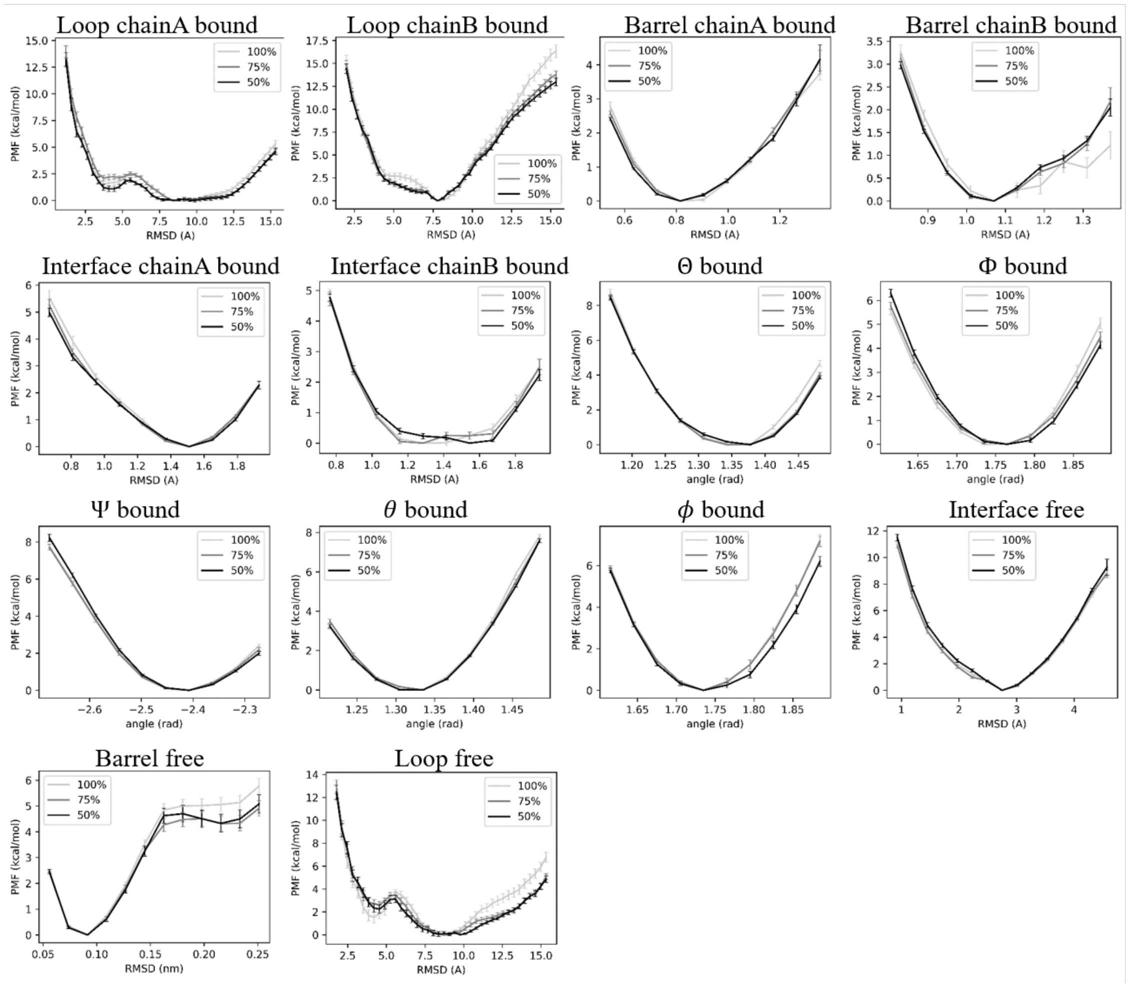

Figure S4: **The PMFs for different fractions of the MD trajectories for D101N E,E(SS), to illustrate convergence.** PMFs constructed with the full, the last 75%, and the last 50% of the REMD-US trajectories are calculated to show the convergence. The PMFs from the top-left to the lower-right are with incremental/decremental restraints, and follows the arrow in Fig. 2 (main text).

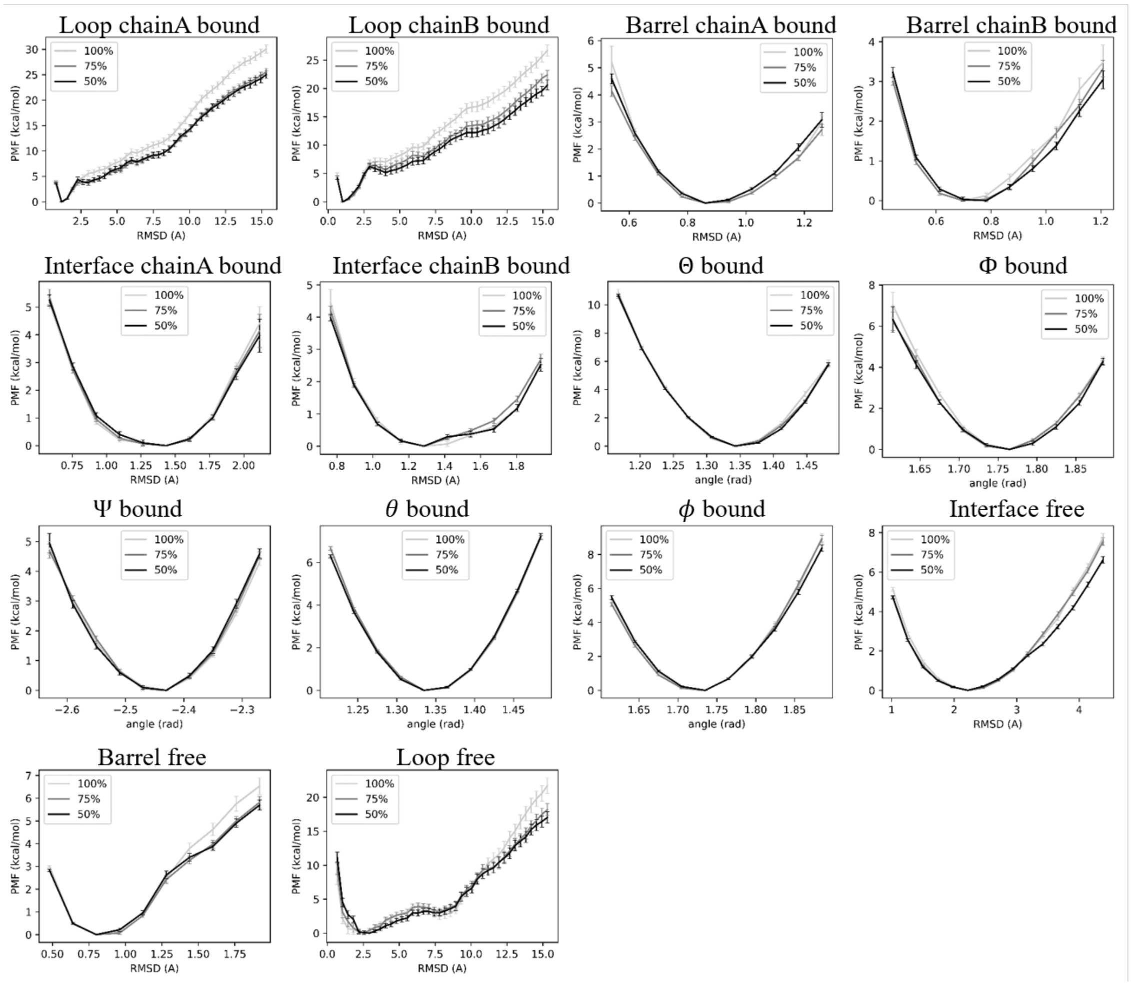

Figure S5: **The PMFs for different fractions of the MD trajectories for WT Cu,Zn(SS), to illustrate convergence.** PMFs constructed with the full, the last 75%, and the last 50% of the REMD-US trajectories are calculated to show the convergence. The PMFs from the top-left to the lower-right are with incremental/decremental restraints, and follows the arrow in Fig. 2 (main text).

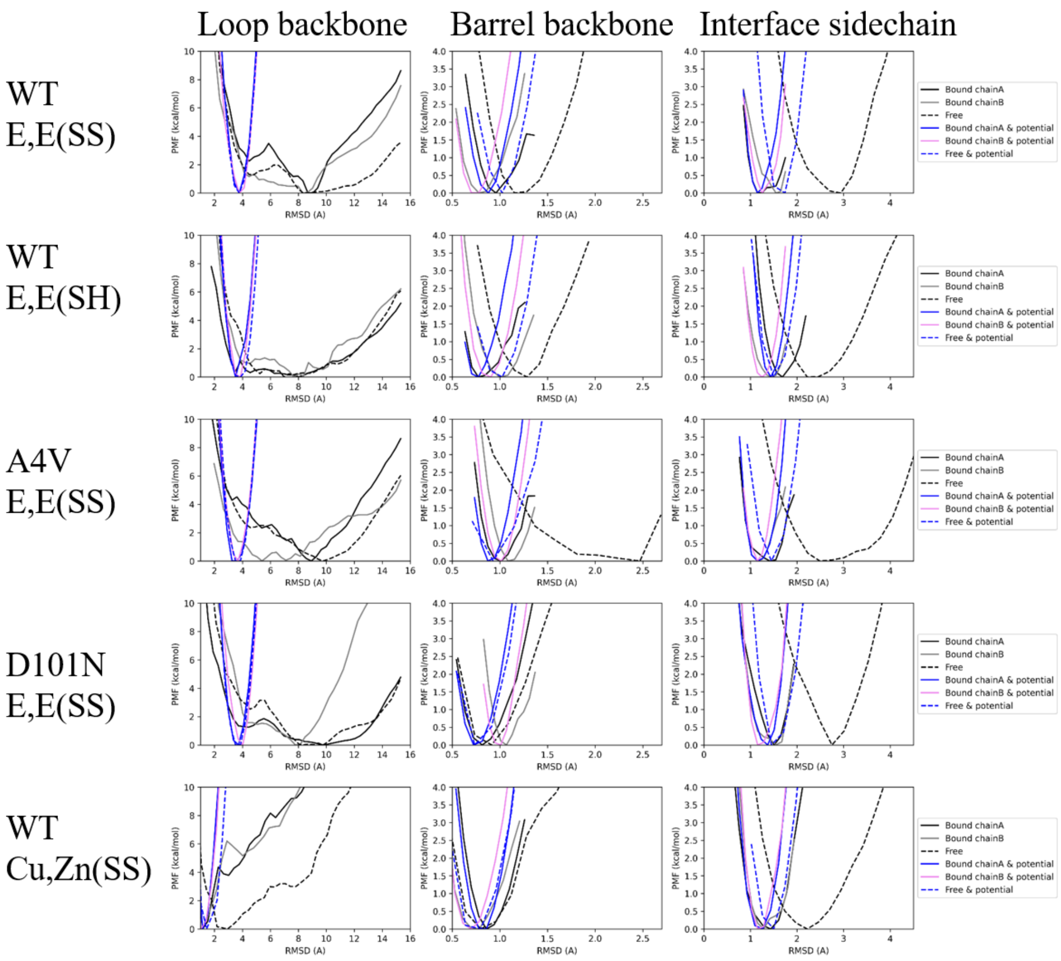

Figure S6: This plot provides additional information to Fig. 3 (main text): Here, the restraint potentials are added on each PMFs to give three additional PMFs in blue in each plot.

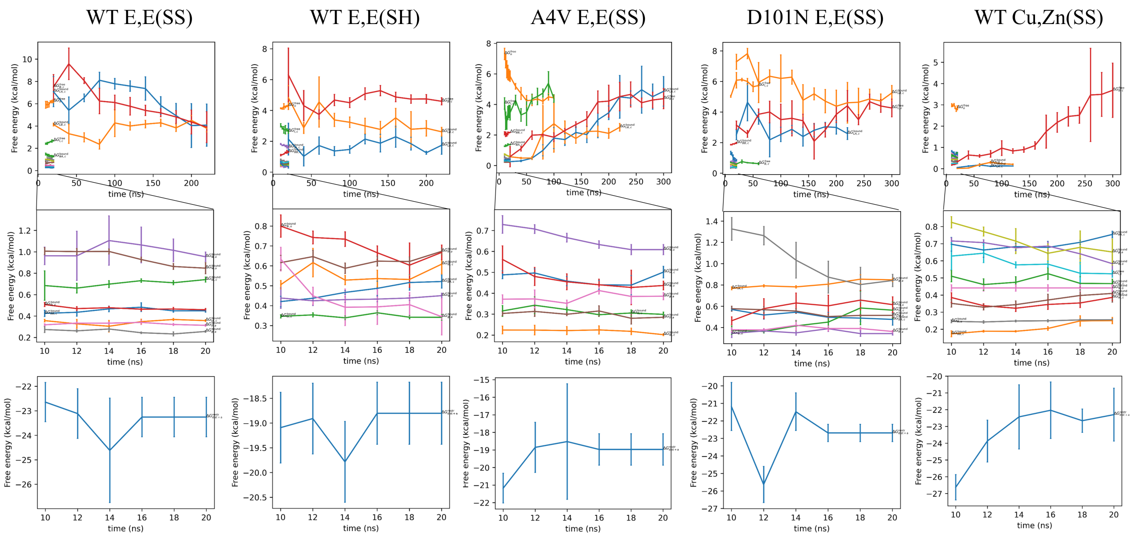

Figure S7: The convergence of each free energy contribution. The free energy values and errors are evaluated with accumulated simulation trajectories.

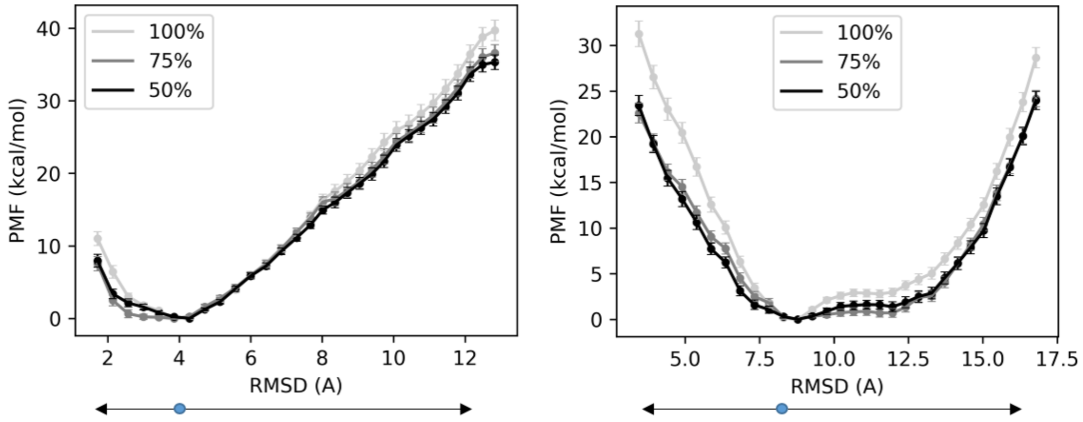

Figure S8: Dimer Loop chain A PMF constructed by umbrella sampling with initial configurations RMSD-steered solely from one of two equilibrated structures. Left panel: The initial configurations are steered from the RMSD∼ 4Å equilibrated structure. Right panel: The initial configurations are steered from the RMSD∼ 8Å equilibrated structure. The schematic arrows below the x-axis in each panel indicate the process of RMSD steering from equilibrated structures (blue dots) where the initial configurations are sampled. The two PMFs are significantly different and do not converge on the time scales of our simulations. For this reason, we did not use either of these seeding methods, but rather mixed initial conditions from both small and large values of RMSD.
